# Global burden of rheumatic heart disease: trends from 1990 to 2019

**DOI:** 10.1186/s13075-022-02829-3

**Published:** 2022-06-11

**Authors:** Zejin Ou, Danfeng Yu, Yuanhao Liang, Jinhua Wu, Huan He, Yongzhi Li, Wenqiao He, Yuhan Gao, Fei Wu, Qing Chen

**Affiliations:** 1 Department of Central Laboratory, Guangzhou Twelfth People’s Hospital, Guangzhou, China; 2 Key Laboratory of Occupational Environment and Health, Guangzhou Twelfth People’s Hospital, Guangzhou, China; 3 Department of MICU, Guangdong Women and Children Hospital, Guangzhou, China; 4 Guangdong Provincial Key Laboratory of Tropical Disease Research, Department of Epidemiology, School of Public Health, Southern Medical University, Guangzhou, 510515 China; 5 Department of Obstetrics, Guangdong Women and Children Hospital, Guangzhou, China

**Keywords:** Rheumatic heart disease, Global Burden of Disease, Age-standardized rate, Estimated annual percentage change, Disability-adjusted life years

## Abstract

**Background:**

Rheumatic heart disease (RHD) is a critical public health issue worldwide, and its epidemiological patterns have changed over the decades. This article aimed to estimate the global trends of RHD, and attributable risks from 1990 to 2019.

**Methods:**

Data on RHD burden were explored from the Global Burden of Disease Study 2019. Trends of the RHD burden were estimated using the estimated annual percentage change (EAPC) and age-standardized rate (ASR).

**Results:**

During 1990–2019, increasing trends in the ASR of incidence and prevalence of RHD were observed worldwide, with the respective EAPCs of 0.58 (95% confidence interval [CI] 0.52 to 0.63) and 0.57 (95%CI 0.50 to 0.63). Meanwhile, increasing trends commonly occurred in low and middle Socio-Demographic Index (SDI) regions and countries. The largest increasing trends in the ASR of incidence and prevalence were seen in Fiji, with the respective EAPCs being 2.17 (95%CI 1.48 to 2.86) and 2.22 (95%CI 1.53 to 2.91). However, death and disability-adjusted life years (DALYs) due to RHD showed pronounced decreasing trends of ASR globally, in which the EAPCs were − 2.98 (95%CI − 3.03 to − 2.94) and − 2.70 (95%CI − 2.75 to − 2.65), respectively. Meanwhile, decreasing trends were also observed in all SDI areas and geographic regions. The largest decreasing trends of death were observed in Thailand (EAPC = − 9.55, 95%CI − 10.48 to − 8.61). Among the attributable risks, behavioral risk-related death and DALYs caused by RHD had pronounced decreasing trends worldwide and in SDI areas.

**Conclusions:**

Pronounced decreasing trends of death and DALYs caused by RHD were observed in regions and countries from 1990 to 2019, but the RHD burden remains a substantial challenge globally. The results would inform the strategies for more effective prevention and control of RHD.

**Supplementary Information:**

The online version contains supplementary material available at 10.1186/s13075-022-02829-3.

## Introduction

Rheumatic heart disease (RHD) is a common acquired heart disease caused by a prior group A streptococcus (GAS) infection. Due to the high risks of premature morbidity, mortality, and disability, RHD remains a critical public health issue worldwide, particularly in many low-income countries [1, 2]. Meanwhile, advances in diagnosis and surgery had promoted the changes in the epidemiological pattern of RHD. Therefore, tracking the temporal trends of RHD burden is necessary for health strategies.

Over the past decades, the RHD control had achieved remarkable progress with the advancements in socioeconomic conditions and health care worldwide [3–5]. The Global Burden of Disease (GBD) Study report revealed that 33.4 million RHD cases, and caused 319,400 deaths globally in 2015, and the age-standardized mortality decreased by 47.8% from 1990 to 2015 [6]. A pronounced decrease in RHD death was reported in the Americas, with the years of life lost (YLLs) fell 88.4/100,000 in 1990 to 38.2/100,000 in 2019 [7]. Despite these improvements, the RHD burden remains a substantial challenge in many regions. A high prevalence of RHD existed among children and young people in many low-income countries, where existed poverty, malnutrition, and inadequate health resource [8–10]. RHD is an important cause of cardiovascular death and disability in low- and middle-income regions, including South Asia, Sub-Saharan Africa, and the Pacific islands [11–13]. In high-income countries, the epidemiology and management of RHD continue to be impacted by the infections of acute rheumatic fever (ARF) in the indigenous populations [14] and the immigrants from high-risk settings [15, 16]. In 2012, the World Health Organization (WHO) and World Heart Federation (WHF) launched a program to reduce 25% of death due to ARF and RHD among the people aged < 25 years by 2025 [17].

The GBD groups estimated and tracked the burden of diseases, injuries, and risk factors worldwide over time, which could inform public health strategies. Therefore, this article aimed to track the global, regional, and national trends of RHD from 1990 to 2019 using the updated data derived from the GBD Study.

## Methods

### Data source

Data on RHD were collected from the GBD Study 2019 using the Global Health Data Exchange (GHDx) query tool (http://ghdx.healthdata.org/gbd-results-tool). The RHD burden includes incidence, prevalence, death, and disability-adjusted life years (DALYs), which were extracted for sexes, age, geographic regions, and countries from 1990 to 2019. The data was available in 21 geographic regions and 204 countries/territories worldwide. The Socio-Demographic Index (SDI) is a summary measure reflecting the average of the incomes per capita, fertility rates, and educational attainment in regions and countries. The value of SDI ranges from 0 to 1, in which 1 means the highest level of average per capita incomes, educational opportunities, and fertility rates, but 0 means the lowest level. For example, the SDI varied from 0.081 in Somalia to 0.929 in Switzerland in 2019. According to the value of SDI, these countries and regions were classified into low, low-middle, middle, high-middle, and high. Data on the Human Development Index (HDI) was obtained from the United Nations Development Program (http://hdr.undp.org/en/data).

The GBD Study summarized and quantified the risks and exposures with the data derived from 46,749 cohort studies, randomized controlled trials, civil surveys, and other sources [18]. Three categorized risks related to death and DALYs due to RHD for the GBD estimates were available, including metabolic, behavioral, and environmental/occupational.

### Statistical analysis

In terms of discrepancy in the age structure of multiple populations over time, age-standardized is a necessary and representative index. The age-standardized rate (ASR) is calculated by using the following formula:

$$\mathrm{ASR}=\frac{\sum_{\overset{A}{i=1}}{a}_i{w}_i}{\sum_{\overset{A}{i=1}}{w}_i}\times 100,000$$

where *a*_*i*_ is the age-specific rate in the *i*th age group, *w* is the number of the weight (people) in the corresponding *i*th age group among the selected standard population, and *A* is the number of age groups.

The estimated annual percentage change (EAPC) is a reliable and widely accepted method to quantify the trends in ASR [19, 20]. A regression line is fitted to the natural logarithm of ASR, with the equation of *y* = *α* + *βx* + *ε*, where *y* = ln (ASR) and *x* = calendar year. EAPC then is calculated with the equation of 100 × (exp(*β*) − 1), and its 95% confidence interval (CI) was estimated using the linear regression model. An increasing trend of ASR is determined if both the EAPC value and its 95%CI > 0. A decreasing trend of ASR is determined if both the EAPC value and 95%CI < 0; others mean the ASR being stable over time. In order to explore the influential factors of EAPC, the relationship between EAPC and ASR in 1990, and between EAPC and HDI in 2019 were investigated using a Pearson correlation analysis. All data were analyzed using R v3.6.2 (R Institute for Statistical Computing, Vienna, Austria). A *p*-value of less than 0.05 was considered to be statistically significant.

## Results

### Trends in incidence of RHD

**Fig. 1:**
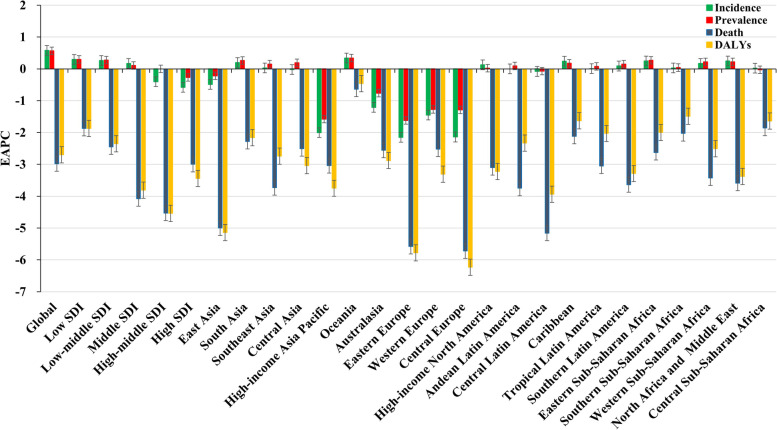
The trends in incidence, prevalence, death, and DALYs of RHD at the global and regional levels, 1990–2019. RHD, rheumatic heart disease; SDI, sociodemographic index; DALYs, disability-adjusted life years

**Fig. 2:**
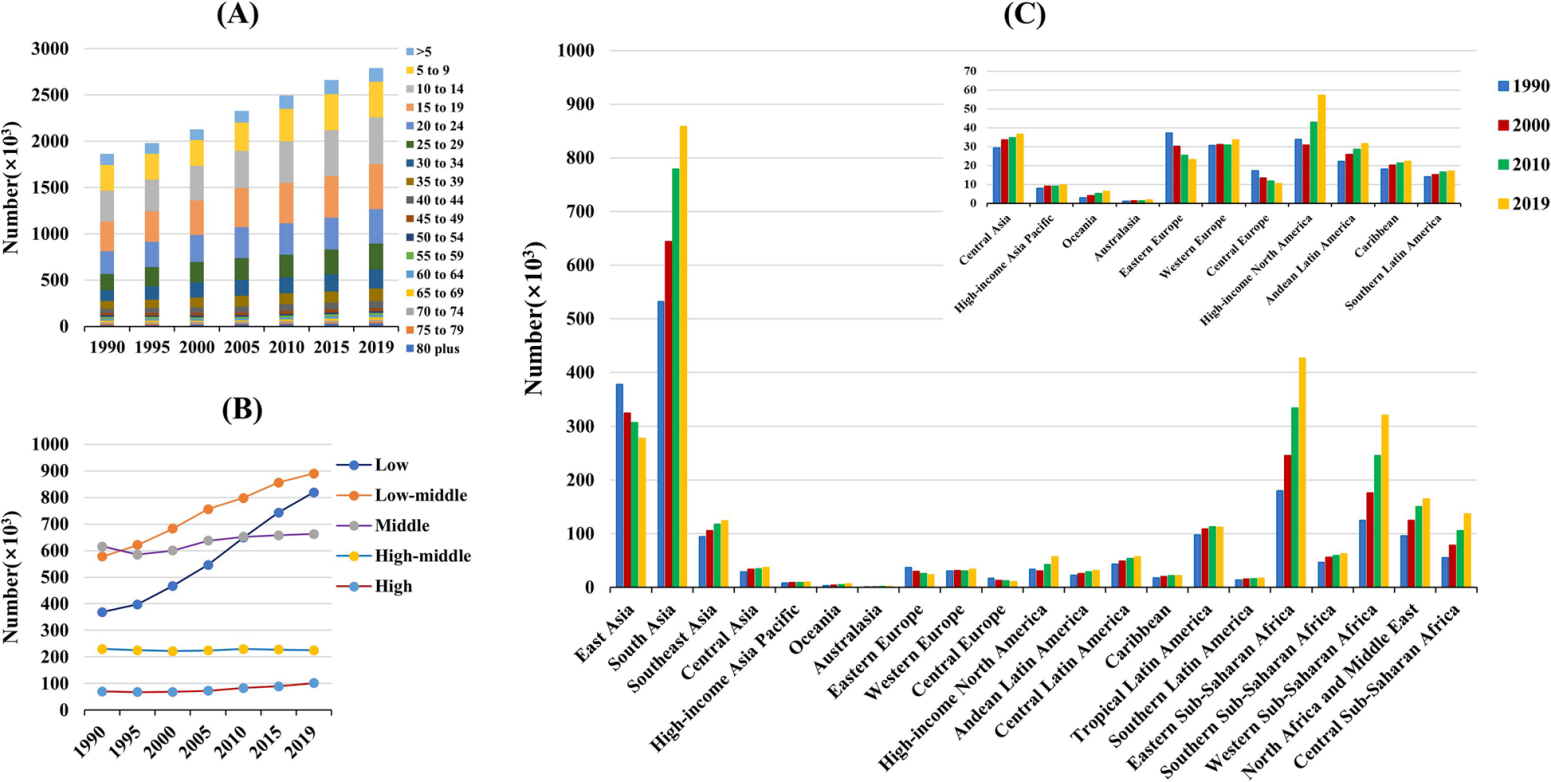
The incident number of RHD in age groups, and in SDI areas and geographic regions, 1990-2019. The incident number in age groups (**A**), SDI areas (**B**), and geographical regions (**C**), respectively. RHD, rheumatic heart disease; SDI, sociodemographic index

**Fig. 3:**
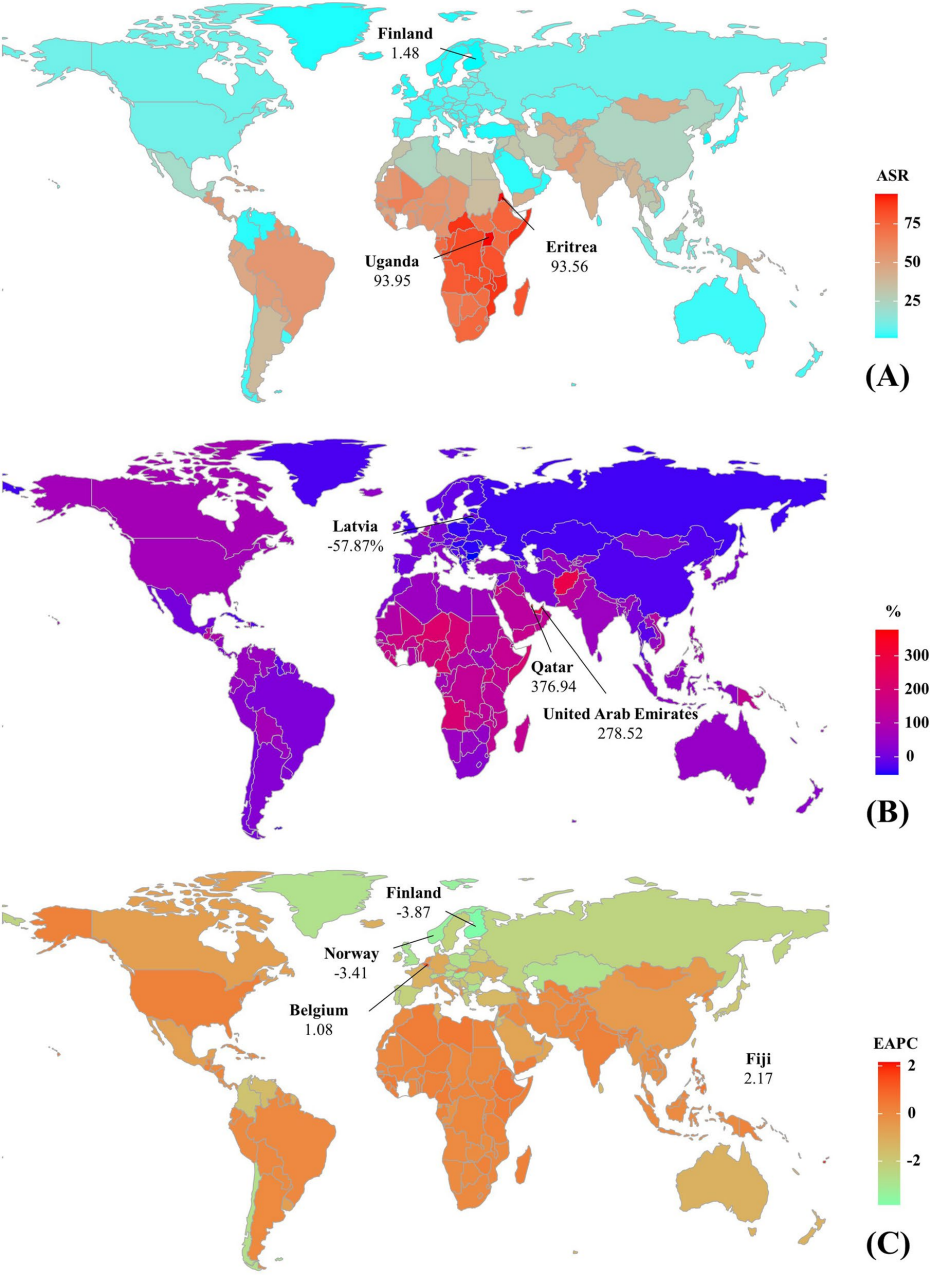
The ASRs, percentage changes, and EAPCs of RHD incidence at the national level, 1990–2019. **A** The ASRs in 2019. **B** The percentage changes in the number between 2000 and 2019. **C** The EAPCs in countries/territories. Countries/territories with an extreme value were annotated. RHD, rheumatic heart disease; ASR, age-standardized rate; EAPC, estimated annual percentage change

**Fig. 4:**
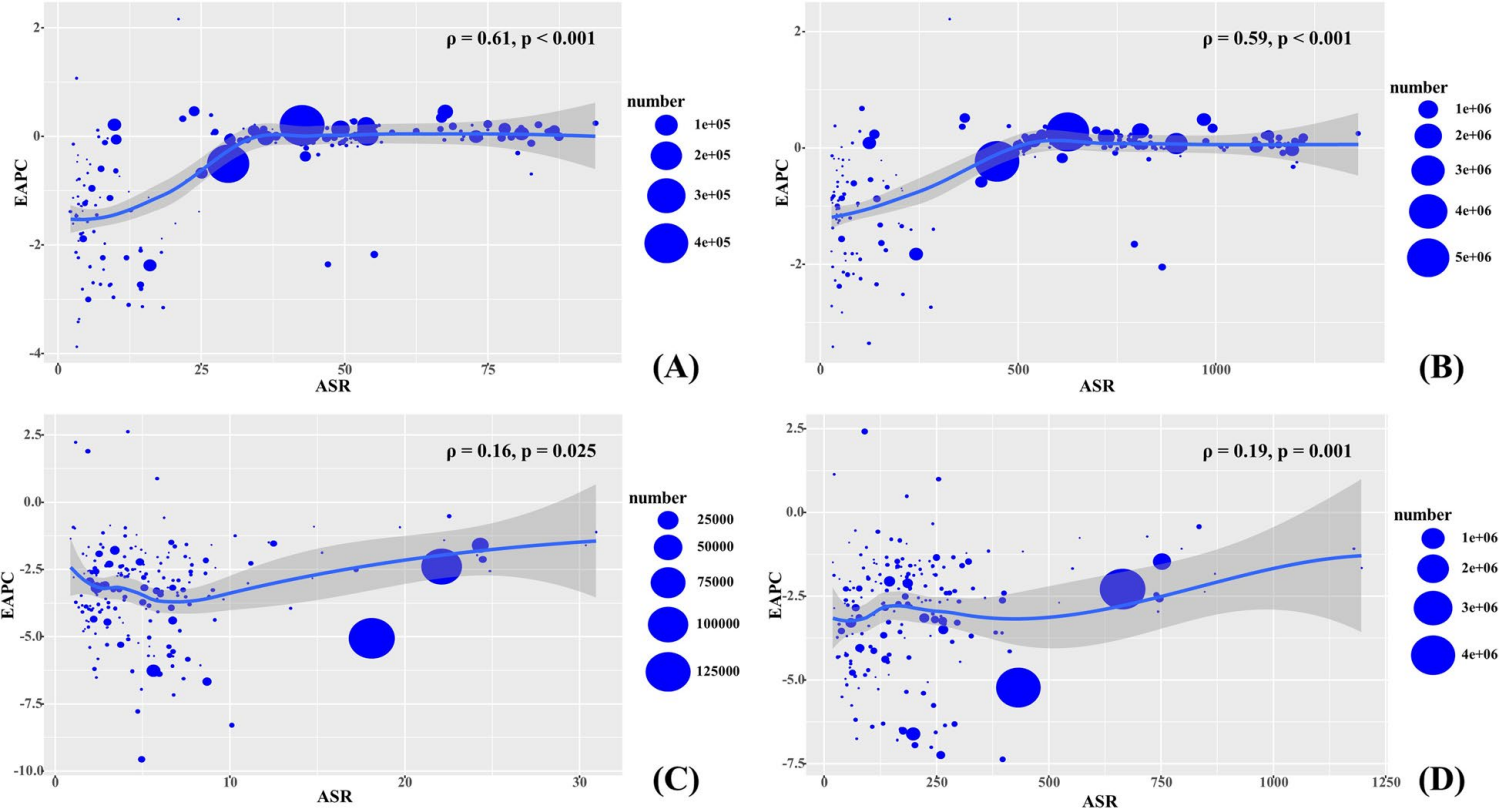
The association between EAPCs and ASRs in 1990 of RHD at the national level. The EAPCs of incidence (**A**), prevalence (**B**), death (**C**), and DALYs (**D**) had positive associations with ASR in 1990. The association was calculated with Pearson correlation analysis. The size of the circle increased with the corresponding numbers in 1990. RHD, rheumatic heart disease; EAPC, estimated annual percentage change; ASR, age-standardized rate; DALYs, disability-adjusted life years

**Fig. 5:**
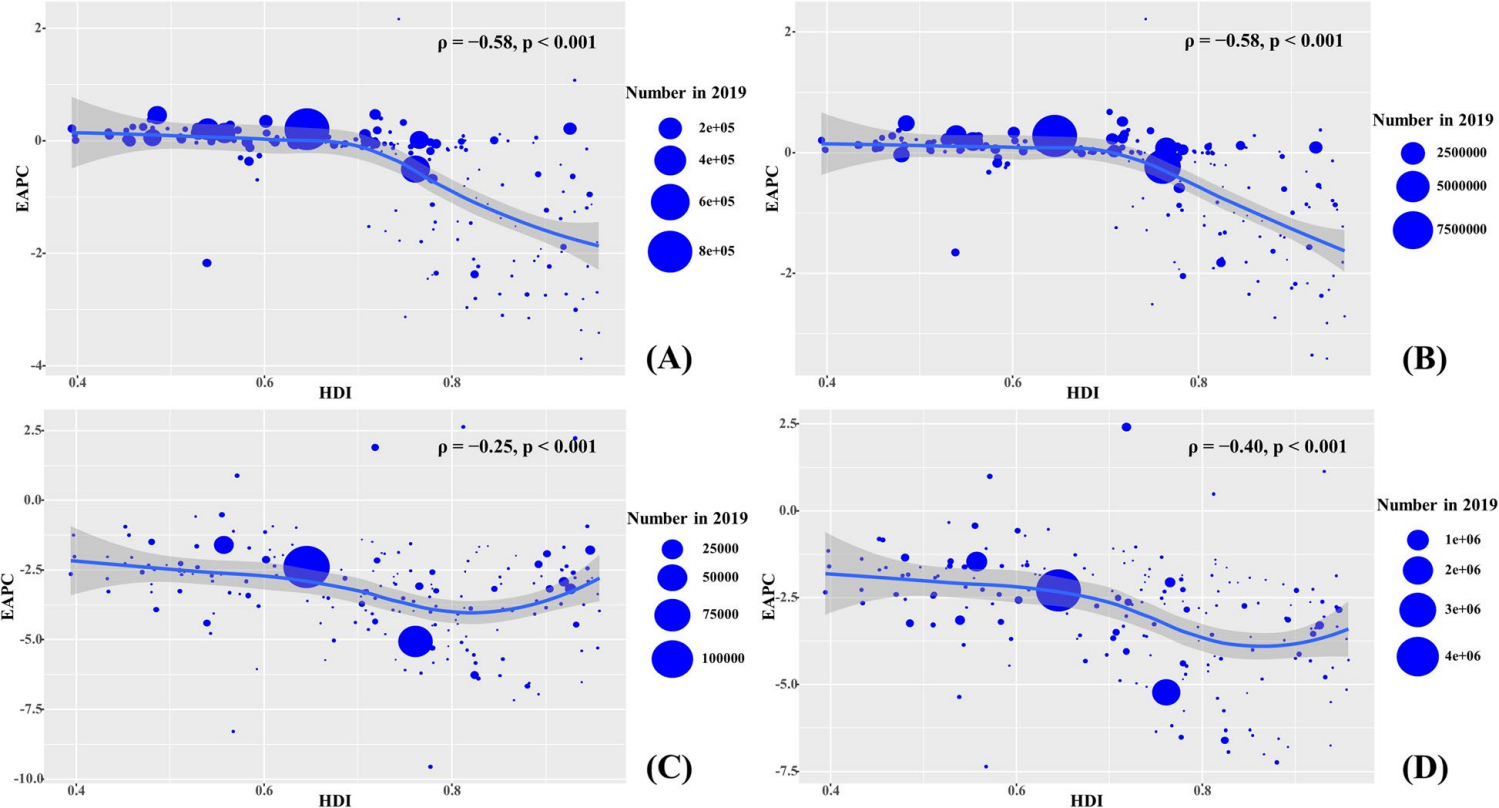
The association between EAPCs of RHD and HDI in 2019 at the national level. The EAPCs of incidence (**A**), prevalence (**B**), death (**C**), and DALYs (**D**) had negative associations with HDI in 2019. The association was calculated with Pearson correlation analysis. The size of the circle increased with the corresponding numbers in 2019. RHD, rheumatic heart disease; EAPC, estimated annual percentage change; ASR, age-standardized rate; DALYs, disability-adjusted life years

Globally, the incident number of RHD was about 2.79 million in 2019, with an increase of 49.70% since 1990. The overall age-standardized incidence rate (ASIR) was 37.40/100,000 in 2019, and increased with an annual average 0.58% from 1990 to 2019 (EAPC = 0.58, 95%CI 0.52 to 0.63) (Table 1; Fig. 1). Compared to males, females had higher incident numbers, while showing a lower increasing trend of ASIR (Table 1). The highest incident number of RHD was observed in the age group of 10–14 years in 2019, and decreasing percent changes in number only occurred in those aged 50–54 years from 1990 to 2019 (Additional file 1: Table S1; Fig. 2A). Among the SDI areas, a pronounced increasing trend of ASIR was seen in the low SDI area (EAPC = 0.30, 95%CI 0.24 to 0.36). In contrast, decreasing trends occurred in high-middle and high SDI areas, particularly the latter (EAPC = − 0.58, 95%CI − 0.74 to − 0.41). In terms of regions, the largest percent increase in incident number was found in western Sub-Saharan Africa (156.05%), while the largest decrease was in Central Europe (− 39.78%). The increasing trends of ASIRs appeared in nine regions, particularly Oceania (EAPC = 0.34, 95%CI 0.27 to 0.40). Conversely, the decreasing trends occurred in seven regions, and the largest one was in Eastern Europe (EAPC = − 2.15, 95%CI − 2.26 to − 2.04), followed by Central Europe and High-income Asia Pacific (Table 1; Figs. 1 and 2B, C). In 2019, the ASIRs were heterogeneous between countries from 1.48/100,000 in Finland to 93.95/100,000 in Uganda. The highest increasing percentages of the incident number were seen in Qatar (376.94%) and United Arab Emirates (278.52%), while the largest decrease was in Latvia (− 57.87%). The increasing trends of ASIR were observed in sixty-five countries/territories, particularly Fiji and Belgium, in which the respective EAPCs were 2.17 (95%CI 1.48 to 2.86) and 1.08 (95%CI 0.76 to 1.39). Conversely, the ASIRs showed decreasing trends in 119 countries/territories, particularly Finland (EAPC = − 3.87, 95%CI − 4.18 to − 3.56), followed by Norway and Singapore (Additional file 1: Table S2; Fig. 3A–C). EAPCs had a positive relationship with the ASRs in 1990 (*ρ* = 0.61, *p* < 0.001; Fig. 4A), and had a negative relationship with HDI (*ρ* = − 0.58, *p* < 0.001; Fig. 5A).

**Table 1:** The percentage changes and EAPCs of incidence and prevalence of RHD from 1990 to 2019 globally and in sexes, SDI areas, and geographic regions

Characteristics	Incidence				Prevalence			
	2019		1990–2019		2019		1990–2019	
	Number, × 10^3^ (95% UI)	ASR/100,000 (95% UI)	Percentage (%)	EAPC (95%CI)	Number, × 10^3^ (95% UI)	ASR/100,000 (95% UI)	Percentage (%)	EAPC (95%CI)
**Overall**	2789.44 (2153.32 to 3454.26)	37.40 (28.6 to 46.74)	49.70	0.58 (0.52 to 0.63)	40,502.34 (32,052.9 to 50,062.43)	513.68 (405.01 to 636.25)	70.49	0.57 (0.50 to 0.63)
**Sex**								
Male	1298.67 (995.19 to 1617.20)	34.33 (26.25 to 42.88)	50.55	0.63 (0.57 to 0.68)	17,983.11 (14,198.98 to 22,406.04)	455.24 (359.15 to 566.15)	68.75	0.59 (0.53 to 0.65)
Female	1490.77 (1159.32 to 1837.88)	40.57 (31.13 to 50.40)	48.97	0.55 (0.49 to 0.6)	22,519.24 (17,895.09 to 27,713.12)	572.23 (450.66 to 705.53)	71.90	0.55 (0.49 to 0.62)
**SDI**								
Low	819.41 (605.20 to 1053.34)	58.86 (44.38 to 74.15)	122.30	0.30 (0.24 to 0.36)	9714.93 (7435.71 to 12,350.76)	886.93 (688.00 to 1106.54)	142.87	0.30 (0.28 to 0.32)
Low-middle	890.24 (670.72 to 1117.22)	46.11 (35.08 to 57.70)	54.03	0.26 (0.22 to 0.30)	12,551.12 (9661.51 to 15,788.80)	687.61 (534.82 to 860.28)	82.78	0.28 (0.25 to 0.31)
Middle	662.47 (509.10 to 826.73)	29.43 (22.42 to 36.89)	7.63	0.17 (0.06 to 0.28)	12,571.43 (9894.03 to 15,706.57)	498.41 (392.05 to 624.2)	44.98	0.11 (0.03 to 0.19)
High-middle	224.99 (190.42 to 265.50)	17.03 (13.68 to 20.67)	− 2.12	− 0.40 (− 0.50 to − 0.30)	4340.13 (3662.38 to 5170.28)	271.54 (224.52 to 329.00)	29.03	0 (− 0.12 to 0.12)
High	101.50 (92.16 to 111.96)	5.86 (5.33 to 6.40)	44.51	− 0.58 (− 0.74 to − 0.41)	1298.83 (1172.66 to 1430.67)	74.97 (68.09 to 82.12)	54.57	− 0.27 (− 0.41 to − 0.13)
**Regions**								
East Asia	277.45 (224.10 to 339.06)	23.85 (18.60 to 29.77)	− 26.49	− 0.49 (− 0.66 to − 0.32)	6155.94 (5006.25 to 7555.64)	387.68 (308.67 to 477.94)	8.51	− 0.22 (− 0.39 to − 0.05)
South Asia	858.39 (633.74 to 1087.91)	18.52 (14.64 to 22.89)	61.33	0.20 (0.15 to 0.24)	12,168.75 (9299.44 to 15,368.47)	284.89 (230.85 to 349.16)	89.87	0.26 (0.22 to 0.31)
Southeast Asia	123.84 (98.21 to 151.61)	40.72 (31.80 to 51.77)	30.58	0.03 (− 0.01 to 0.07)	1995.18 (1608.08 to 2455.92)	587.08 (468.25 to 748.25)	64.83	0.15 (0.11 to 0.19)
Central Asia	36.56 (28.02 to 45.60)	38.30 (29.32 to 47.78)	25.32	− 0.02 (− 0.07 to 0.04)	599.38 (468.44 to 742.53)	623.66 (490.35 to 770.96)	52.28	0.19 (0.17 to 0.21)
High-income Asia Pacific	9.73 (8.26 to 11.26)	6.45 (5.78 to 7.17)	24.21	− 2.00 (− 2.11 to − 1.90)	128.66 (109.07 to 148.55)	93.46 (82.24 to 105.61)	30.03	− 1.58 (− 1.69 to − 1.47)
Oceania	6.30 (4.84 to 8.05)	8.02 (7.03 to 9.07)	111.34	0.34 (0.27 to 0.40)	79.24 (62.04 to 101.49)	135.51 (118.27 to 154.99)	125.21	0.34 (0.27 to 0.41)
Australasia	1.65 (1.45 to 1.88)	2.46 (2.12 to 2.81)	44.19	− 1.21 (− 1.38 to − 1.04)	21.39 (18.22 to 24.87)	34.16 (29.55 to 38.97)	54.60	− 0.76 (− 0.91 to − 0.60)
Eastern Europe	23.02 (19.76 to 26.24)	3.80 (3.37 to 4.31)	− 38.01	− 2.15 (− 2.26 to − 2.04)	435.22 (376.40 to 502.43)	50.71 (42.99 to 58.59)	− 26.82	− 1.62 (− 1.82 to − 1.42)
Western Europe	33.62 (30.17 to 37.43)	3.90 (3.48 to 4.34)	9.84	− 1.45 (− 1.51 to − 1.38)	339.48 (288.52 to 394.43)	39.72 (33.93 to 45.61)	9.96	− 1.27 (− 1.33 to − 1.20)
Central Europe	10.38 (9.18 to 11.62)	27.86 (21.4 to 34.78)	− 39.78	− 2.14 (− 2.28 to − 2.00)	171.07 (149.02 to 195.26)	488.01 (385.13 to 603.26)	− 18.36	− 1.29 (− 1.39 to − 1.18)
High-income North America	57.23 (51.68 to 63.34)	9.35 (8.50 to 10.31)	69.71	0.13 (− 0.13 to 0.40)	723.74 (660.55 to 787.48)	117.54 (107.71 to 127.03)	68.43	0.02 (− 0.16 to 0.21)
Andean Latin America	31.56 (23.71 to 39.73)	49.12 (37.16 to 62.06)	42.21	0 (− 0.01 to 0.01)	527.24 (407.02 to 656.08)	786.82 (612.48 to 977.11)	80.88	0.09 (0.07 to 0.11)
Central Latin America	56.91 (43.98 to 70.34)	47.96 (36.13 to 60.44)	30.51	− 0.08 (− 0.15 to − 0.01)	914.29 (720.26 to 1122.68)	812.15 (628.58 to 1011.81)	64.75	− 0.07 (− 0.13 to − 0.01)
Caribbean	22.11 (16.77 to 27.66)	22.63 (17.40 to 28.00)	22.09	0.24 (0.23 to 0.25)	379.16 (295.53 to 469.69)	353.88 (279.49 to 434.27)	43.02	0.18 (0.18 to 0.18)
Tropical Latin America	111.88 (84.85 to 139.76)	54.01 (40.56 to 68.35)	14.45	0.01 (0 to 0.02)	2165.35 (1688.93 to 2683.95)	917.15 (714.71 to 1141.65)	56.25	0.08 (0.07 to 0.09)
Southern Latin America	17.07 (13.22 to 21.21)	25.61 (19.71 to 31.91)	22.57	0.09 (0.07 to 0.12)	336.60 (267.35 to 414.13)	388.89 (304.75 to 483.49)	45.94	0.15 (0.12 to 0.18)
Eastern Sub-Saharan Africa	426.53 (315.69 to 555.54)	42.98 (32.15 to 54.22)	137.16	0.25 (0.23 to 0.26)	4767.28 (3635.37 to 6158.95)	645.12 (498.23 to 811.68)	150.84	0.27 (0.25 to 0.28)
Southern Sub-Saharan Africa	62.17 (46.40 to 78.75)	81.68 (60.58 to 104.25)	34.04	0.03 (0.01 to 0.04)	898.70 (689.77 to 1145.95)	1195.56 (918.19 to 1511.29)	59.36	0.04 (0.03 to 0.06)
Western Sub-Saharan Africa	320.73 (235.87 to 419.48)	80.98 (60.90 to 103.24)	156.05	0.17 (0.15 to 0.19)	3674.37 (2770.19 to 4722.20)	1174.40 (904.04 to 1475.04)	162.05	0.22 (0.19 to 0.24)
North Africa and Middle East	165.23 (127.31 to 205.82)	72.77 (54.23 to 92.50)	71.49	0.25 (0.20 to 0.31)	2474.18 (1922.46 to 3094.27)	1090.22 (844.68 to 1377.19)	110.92	0.22 (0.17 to 0.27)
Central Sub-Saharan Africa	137.07 (99.45 to 180.49)	56.21 (42.41 to 71.26)	146.19	0.02 (− 0.02 to 0.05)	1547.13 (1157.71 to 1995.34)	839.55 (651.29 to 1057.38)	152.73	− 0.03 (− 0.04 to − 0.01)

*RHD* rheumatic heart disease, *EAPC* estimated annual percentage change, *ASR* age-standardized rate, *CI* confidence interval, *UI* uncertainty interval, *SDI* socio-demographic index

### Trends in prevalence of RHD

During 1990–2019, the global RHD prevalence increased by 70.49% and reached 40.50 million in 2019. The total ASR of prevalence was 513.68/100,000 in 2019, and the overall ASR had an increasing trend (EAPC = 0.57, 95%CI 0.50 to 0.63) (Table 1; Fig. 1). Compared with males, females had a higher prevalence number, while showing a lower increasing trend of ASRs (Table 1). The highest RHD prevalence was observed in the age group of 25–29 years in 2019, and the increasing percentage occurred in all age groups, particularly those aged above 80 years (140.36%) (Additional file 1: Table S1, Fig. S1A). The ASRs of prevalence had increasing trends in low, low-middle, and middle SDI areas, while decreasing in high SDI areas (EAPC = − 0.27, 95%CI − 0.41 to − 0.13). In 21 geographic regions, the highest RHD prevalent number was found in South Asia (12.17 million), and the largest increasing percent occurred in Western Sub-Saharan Africa (162.05%). The increasing trends of ASRs were observed in twelve regions, particularly Oceania (EAPC = 0.34, 95%CI 0.27 to 0.41). However, decreasing trends were found in ten geographic regions, especially in Eastern Europe (EAPC = − 1.62, 95%CI − 1.82 to − 1.42), followed by High-income Asia Pacific and Central Europe (Table 1; Fig. 1, and Additional file 1: Fig. S1B-C). At the national level, the largest increasing prevalence number was observed in the United Arab Emirates (494.57%), whereas the largest decreasing one was in Moldova (− 43.34%). The trends of prevalent ASRs increased in ninety-seven countries/territories, and the most pronounced ones occurred in Fiji (EAPC = 2.22, 95%CI 1.53 to 2.91). However, the trends declined in ninety-one countries/territories, particularly Finland (EAPC = − 3.41, 95%CI − 3.86 to − 2.96), followed by Austria and Singapore (Additional file 1: Table S2, Fig. S2A-C). EAPCs had a positive relationship with the ASRs in 1990 (*ρ* = 0.59, *p* < 0.001; Fig. 4B), and had a negative relationship with HDI (*ρ* = − 0.58, *p* < 0.001; Fig. 5B).

### Trends in death due to RHD

The death number of RHD was about 0.31 million worldwide in 2019, with a decrease of 15.60% since 1990. The overall age-standardized death rate (ASDR) of RHD was 3.85/100,000 in 2019, and presented a decreasing trend during 1990–2019 (EAPC = − 2.98, 95%CI − 3.03 to − 2.94) (Table 2; Fig. 1). Female patients had a higher death number, while showing a larger decreasing trend of ASDR (EAPC = − 3.10, 95%CI − 3.17 to − 3.03) (Table 2). The highest number of RHD prevalence was observed in the age group above 80 years, and the largest decreasing changes in number occurred in those aged under 5 years (− 75.35%) (Additional file 1: Table S1, Fig. S3A). Trends in ASDR of RHD decreased in sexes, SDI areas, and geographic regions from 1990 to 2019, particularly the high-middle SDI areas (EAPC = − 4.53, 95%CI − 4.67 to − 4.39). At the regional level, Central Europe and Eastern Europe had the most pronounced decreasing trends, with the respective EAPCs being − 5.72 (95%CI − 6.14 to − 5.29) and − 5.58 (95%CI − 6.07 to − 5.09) (Table 2; Fig. 1, and Additional file 1: Fig. S3B-C). At the national level, the largest increasing change in the number of RHD was seen in United Arab Emirates (219.33%), whereas the largest decreasing one was in Latvia (− 83.32%). Decreasing trends in ASDR of RHD were observed in 200 countries/territories, particularly Thailand and the Syrian Arab Republic, in which the respective EAPCs were − 9.55 (95%CI − 10.48 to − 8.61) and − 8.28 (95%CI − 9.33 to − 7.22). However, increasing trends were found in only four countries, particularly Georgia and Belgium, with the respective EAPCs being 2.64 (95%CI 2.12 to 3.16) and 2.24 (95%CI 1.70 to 2.77) (Additional file 1: Table S3, Fig. S4A-C). EAPCs had a positive relationship with the ASRs in 1990 (*ρ* = 0.16, *p* = 0.025; Fig. 4C), and had a negative relationship with HDI (*ρ* = − 0.25, *p* < 0.001; Fig. 5C).

**Table 2:** The percentage changes and EAPCs of death and DALYs caused by RHD from 1990 to 2019 globally, and in sexes, SDI areas, and geographic regions

Characteristics	Death				DALYs			
	2019		1990–2019		2019		1990–2019	
	Number, × 10^3^ (95% UI)	ASR (100,000), (95% UI)	Percentage (%)	EAPC (95%CI)	Number, × 10^3^ (95% UI)	ASR (100,000), (95% UI)	Percentage (%)	EAPC (95%CI)
**Overall**	305.65 (259.22 to 340.49)	3.85 (3.29 to 4.29)	− 15.60	− 2.98 (− 3.03 to − 2.94)	10,673.88 (9207.38 to 12,121.61)	132.88 (115.02 to 150.34)	− 18.94	− 2.70 (− 2.75 to − 2.65)
**Sex**								
Male	131.72 (113.45 to 159.9)	3.62 (3.14 to 4.37)	− 13.40	− 2.86 (− 2.88 to − 2.83)	4833.46 (4121.95 to 5729.11)	123.51 (105.78 to 145.93)	− 15.91	− 2.53 (− 2.56 to − 2.49)
Female	173.93 (140.65 to 208.09)	4.06 (3.29 to 4.85)	− 17.20	− 3.10 (− 3.17 to − 3.03)	5840.42 (4813.98 to 6963.02)	141.99 (117.47 to 168.62)	− 21.30	− 2.85 (− 2.92 to − 2.77)
**SDI**								
Low	47.51 (39.42 to 55.97)	8.50 (6.99 to 10.15)	23.75	− 1.87 (− 1.95 to − 1.79)	2191.85 (1819.05 to 2580.12)	275.50 (228.02 to 324.57)	25.52	− 1.87 (− 1.96 to − 1.79)
Low-middle	113.46 (85.23 to 135.03)	8.35 (6.34 to 9.95)	7.07	− 2.45 (− 2.54 to − 2.36)	4268.61 (3371.73 to 5010.50)	266.56 (207.28 to 313.76)	− 0.26	− 2.35 (− 2.44 to − 2.26)
Middle	82.95 (70.81 to 93.79)	3.72 (3.17 to 4.23)	− 32.65	− 4.08 (− 4.14 to − 4.02)	2773.06 (2416.94 to 3165.51)	112.05 (97.74 to 127.84)	− 37.29	− 3.81 (− 3.87 to − 3.74)
High-middle	36.96 (33.18 to 40.19)	1.90 (1.70 to 2.07)	− 45.08	− 4.53 (− 4.67 to − 4.39)	1021.63 (914.61 to 1141.70)	56.12 (49.74 to 63.59)	− 51.67	− 4.54 (− 4.67 to − 4.41)
High	24.62 (20.84 to 27.18)	1.13 (0.98 to 1.24)	− 9.50	− 3.00 (− 3.20 to − 2.81)	411.43 (372.56 to 448.62)	22.83 (20.92 to 24.88)	− 31.48	− 3.44 (− 3.70 to − 3.17)
**Regions**								
East Asia	72.40 (60.32 to 83.88)	4.01 (3.34 to 4.63)	− 46.40	− 5.00 (− 5.12 to − 4.89)	1778.09 (1498.96 to 2041.20)	94.12 (79.47 to 108.54)	− 56.56	− 5.14 (− 5.24 to − 5.04)
South Asia	155.98 (119.89 to 187.68)	1.30 (1.11 to 1.48)	25.40	− 2.28 (− 2.43 to − 2.13)	5755.42 (4558.37 to 6820.45)	58.53 (50.19 to 67.44)	12.95	− 2.16 (− 2.29 to − 2.03)
Southeast Asia	7.77 (6.69 to 8.85)	16.06 (10.07 to 24.64)	− 26.47	− 3.73 (− 3.82 to − 3.63)	397.46 (338.91 to 459.41)	627.42 (404.06 to 918.03)	− 20.45	− 2.74 (− 2.79 to − 2.69)
Central Asia	3.29 (2.83 to 3.77)	4.16 (3.64 to 4.69)	− 16.82	− 2.51 (− 2.83 to − 2.19)	147.63 (125.87 to 172.89)	161.24 (138.56 to 187.45)	− 20.04	− 3.04 (− 3.40 to − 2.68)
High-income Asia Pacific	5.11 (3.84 to 5.9)	1.30 (1.14 to 1.47)	51.09	− 3.04 (− 3.10 to − 2.98)	62.42 (51.83 to 69.51)	33.27 (29.12 to 37.55)	− 14.85	− 3.75 (− 3.82 to − 3.67)
Oceania	1.50 (0.94 to 2.24)	1.32 (1.16 to 1.49)	84.17	− 0.64 (− 0.74 to − 0.55)	74.50 (48.60 to 107.66)	44.73 (39.39 to 50.56)	85.86	− 0.46 (− 0.57 to − 0.35)
Australasia	0.61 (0.52 to 0.70)	0.81 (0.64 to 0.93)	6.30	− 2.56 (− 2.79 to − 2.33)	11.47 (10.17 to 12.90)	13.20 (11.53 to 14.46)	− 17.41	− 2.88 (− 3.10 to − 2.67)
Eastern Europe	4.36 (3.83 to 4.91)	1.17 (1.01 to 1.33)	− 68.08	− 5.58 (− 6.07 to − 5.09)	139.01 (122.23 to 156.95)	26.06 (23.14 to 29.22)	− 70.49	− 5.78 (− 6.27 to − 5.29)
Western Europe	16.19 (13.65 to 18.14)	1.47 (1.27 to 1.63)	− 7.84	− 2.52 (− 2.65 to − 2.39)	229.98 (204.56 to 251.23)	25.02 (22.79 to 27.15)	− 34.82	− 3.31 (− 3.49 to − 3.12)
Central Europe	2.76 (2.42 to 3.11)	2.18 (1.81 to 2.57)	− 68.67	− 5.72 (− 6.14 to − 5.29)	63.34 (55.48 to 71.10)	60.62 (49.8 to 73.88)	− 75.80	− 6.23 (− 6.71 to − 5.76)
High-income North America	6.79 (5.93 to 7.42)	1.00 (0.89 to 1.09)	− 18.80	− 3.10 (− 3.45 to − 2.76)	141.20 (126.97 to 157.25)	23.79 (21.53 to 26.44)	− 27.34	− 3.22 (− 3.62 to − 2.82)
Andean Latin America	0.63 (0.51 to 0.78)	2.46 (1.78 to 3.44)	− 15.50	− 3.75 (− 3.85 to − 3.65)	42.24 (31.31 to 56.12)	142.02 (105.28 to 189.56)	4.92	− 2.33 (− 2.45 to − 2.21)
Central Latin America	1.66 (1.42 to 1.92)	1.13 (0.90 to 1.39)	− 40.37	− 5.16 (− 5.38 to − 4.95)	91.82 (72.94 to 116.67)	67.40 (50.19 to 88.85)	− 31.38	− 3.94 (− 4.19 to − 3.69)
Caribbean	1.22 (0.89 to 1.71)	0.70 (0.60 to 0.81)	− 13.98	− 2.12 (− 2.27 to − 1.96)	68.58 (50.94 to 90.78)	36.38 (29.05 to 46.02)	− 13.64	− 1.63 (− 1.80 to − 1.47)
Tropical Latin America	2.77 (2.55 to 2.96)	1.15 (1.06 to 1.23)	− 11.62	− 3.05 (− 3.15 to − 2.94)	188.94 (147.04 to 244.65)	79.03 (61.33 to 102.28)	− 5.98	− 2.03 (− 2.07 to − 2.00)
Southern Latin America	1.86 (1.54 to 2.20)	1.65 (1.35 to 2.00)	− 26.33	− 3.64 (− 3.76 to − 3.53)	46.52 (38.52 to 55.80)	67.07 (54.15 to 82.44)	− 32.79	− 3.29 (− 3.42 to − 3.16)
Eastern Sub-Saharan Africa	4.69 (3.86 to 5.57)	11.18 (8.62 to 13.40)	− 6.58	− 2.63 (− 2.72 to − 2.55)	423.97 (319.46 to 558.81)	348.46 (272.37 to 412.24)	28.13	− 2.00 (− 2.07 to − 1.94)
Southern Sub-Saharan Africa	1.37 (1.15 to 1.64)	3.96 (2.44 to 6.40)	− 4.78	− 2.03 (− 2.38 to − 1.68)	99.85 (78.75 to 123.99)	161.30 (114.77 to 227.69)	5.73	− 1.49 (− 1.71 to − 1.26)
Western Sub-Saharan Africa	5.05 (4.04 to 6.49)	2.45 (2.01 to 2.95)	− 19.19	− 3.43 (− 3.58 to − 3.28)	376.69 (285.46 to 495.50)	122.36 (96.10 to 152.73)	20.62	− 2.51 (− 2.65 to − 2.36)
North Africa and Middle East	7.40 (5.90 to 9.15)	2.18 (1.86 to 2.56)	− 25.14	− 3.59 (− 3.73 to − 3.44)	379.36 (305.22 to 469.08)	128.96 (103.43 to 158.74)	− 27.42	− 3.38 (− 3.51 to − 3.25)
Central Sub-Saharan Africa	2.25 (1.40 to 3.63)	2.42 (1.97 to 3.01)	22.98	− 1.86 (− 2.07 to − 1.65)	155.37 (110.99 to 213.80)	105.13 (82.44 to 132.56)	49.49	− 1.64 (− 1.78 to − 1.50)

*RHD* rheumatic heart disease, *DALYs* disability-adjusted life years, *EAPC* estimated annual percentage change, *ASR* age-standardized rate, *CI* confidence interval, *UI* uncertainty interval, *SDI* socio-demographic index

### Trends in DALYs due to RHD

Globally, the number of DALYs due to RHD was 10.67 million in 2019, with a decrease of 18.94% since 1990. The total ASR of DALYs was 132.88/100,000 in 2019, and decreasing trend of ASR was observed worldwide from 1990 to 2019 (EAPC = − 2.70, 95%CI − 2.75 to − 2.65) (Table 2; Fig. 1). During 1990–2019, the percentage of DALYs number declined in all age groups under 80 years, particularly those under 5 years (− 73.74%) (Additional file 1: Table S1, Fig. S5A). Decreasing trends in ASRs of DALYs were seen in sexes, SDI areas, and geographic regions, particularly in the high-middle SDI area and Central Europe, in which the respective EAPCs were − 4.54 (95%CI − 4.67 to − 4.41) and − 6.23 (95%CI − 6.71 to − 5.76) (Table 2; Fig. 1, and Additional file 1: Fig. S5B-C). Among 204 countries/territories, the largest increasing percentage of DALY number was seen in United Arab Emirates (254.82%), whereas the most pronounced decreasing one was in Latvia (− 84.03%). Decreasing trends in ASRs of DALYs were seen in 200 countries/territories, particularly the Syrian Arab Republic (EAPC = − 7.36, 95%CI − 8.32 to − 6.38), followed by Poland and Latvia. However, increasing trends occurred in four countries, particularly the Philippines and Belgium, in which the respective EAPCs were 2.42 (95%CI 1.70 to 3.14) and 1.14 (95%CI 0.75 to 1.54) (Additional file 1: Table S3, Fig. S6A-C). EAPCs had a positive relationship with ASRs in 1990 at the national level (*ρ* = 0.19, *p* = 0.001; Fig. 4D), and had a negative relationship with HDI (*ρ* = − 0.40, *p* < 0.001; Fig. 5D).

### Trends in attributable risk-related death and DALYs due to RHD

All the three attributable risk-related death caused by RHD showed decreasing trends worldwide from 1990 to 2019. Behavioral risk-related death due to RHD had the most significant decrease in number (− 37.73%) and the ASDR (EAPC = − 3.99, 95%CI − 4.06 to − 3.92) (Additional file 1: Table S4, Fig. S7A-B). Similar decreasing trends were observed in both sexes, particularly behavioral risk-related death in females (EAPC = − 4.50, 95%CI − 4.59 to − 4.41) (Additional file 1: Table S5, Fig. S8A-D). In SDI areas, the risk-related death due to RHD showed sharp decreasing trends in middle and high-middle SDI areas, particularly behavioral risk-related death in the high-middle SDI area, with the EAPC being − 5.28 (95%CI − 5.40 to − 5.16) (Additional file 1: Table S6).

The three attributable risk-related DALYs due to RHD showed pronounced decreasing trends globally from 1990 to 2019, particularly behavioral risk-related DALYs (EAPC = − 3.83, 95%CI − 3.91 to − 3.76) (Additional file 1: Table S4; Fig. S7C-D). Compared with males, more pronounced decreasing trends of risk-related DALYs due to RHD were observed in females, particularly the behavioral risk-related one (EAPC = − 4.39, 95%CI − 4.50 to − 4.28) (Additional file 1: Table S5, Fig. S9A-D). Meanwhile, the strong decreasing trends were also seen in all SDI areas, particularly environmental/occupational risk- and behavioral risk-related DALYs in the high-middle SDI areas, with the respective EAPCs being − 5.35 (95%CI − 5.56 to − 5.15) and − 5.09 (95%CI − 5.21 to − 4.97) (Additional file 1: Table S6).

## Discussion

The limitations should be interpreted in the present study. First, the GBD estimates depended on the quality and quantity of the data. Potential bias derived from the miscoding and misclassification of disease probably impacted the accuracy and robustness of the results. Second, the diagnosis and detection of RHD had refined across countries and over time, which was the leading source of potential bias. Third, age is an essential factor in RHD, but the trends were described using the percentage changes in age groups.

In this work, pronounced decreasing trends of death and DALYs due to RHD were demonstrated worldwide, and in most SDI areas, geographic regions, and countries during the period of 1990–2019, which was similar to the GBD Study of RHD [6]. These achievements were probably due to the effective strategies in the past decades, such as improved health resources, effective prevention and control, and international cooperation [17, 21, 22]. For example, progress in the application of benzathine penicillin, cardiac surgery, and diagnosis and management of ARF and RHD were observed in Africa from 1961 to 2018 [23]. Meanwhile, the low-cost telehealth model facilitated the availability of echocardiography to RHD patients in low- and middle-income countries [24]. In Central Europe and Eastern Europe, pronounced decreasing trends of incidence and prevalence probably were attributable to the construction of the health care system, particularly for children and adolescents [25]. At the national level, Thailand showed the most prominent decreasing trend of death caused by RHD, probably due to economic development and urbanization [26]. In the Syrian Arab Republic, the decreasing trends were probably related to the displacement of refugees due to local unrest [27]. However, increasing trends of death and DALYs were observed in Zimbabwe, Belgium, and the Philippines. Unfavorable trends occurred in Zimbabwe, where the prevalence of heart failure caused by RHD in children and adolescents showed the highest proportion (74%) worldwide [28]. The increasing trends in Belgium were associated with the epidemiologic features of GAS infections, which were predominantly pediatric pharyngitis (88%) at the earlier mean age [29]. In recent years, the GAS infections rapidly increased in homeless populations and intravenous drug users in European cities [30, 31]. The high morbidity and mortality of the GAS infection were found in the homeless persons in Brussels, Belgium [32]. There certainly existed other potential risk factors; only three categorized risks associated with death and DALYs due to RHD were analyzed in the present work. Metabolic risk-related death and DALYs probably benefited from the management of blood pressure in hypertension and cardiovascular patients [33, 34].

However, increasing trends in incidence and prevalence of RHD were observed worldwide from 1990 to 2017. The disease burden caused by GAS continues to be reported worldwide, particularly in low-resource settings [35]. RHD is a neglected chronic disease in many regions and has a high risk in young people after GAS infection [36]. Meanwhile, poor compliance to penicillin G prophylaxis was widespread in high-risk areas, and as much as 70% of patients in Uganda were non- or poorly compliant [37]. Educational programs for this disease were generally lacking in these areas and countries [38, 39]. Incidence and prevalence of RHD had pronounced increasing trends in Oceania, where the contributable factors included poverty and poor medical resources [40], and the genetic susceptibility to RHD [41]. Among the 204 countries/territories, the most prominent decreasing trends in incidence and prevalence were observed in Finland, Norway, Singapore, and Austria, where there have a sound health system, adequate medical resources, and high social welfare, which also explained why EAPCs had a negative relationship with HDI. However, considerably increasing trends of incidence and prevalence caused by RHD were seen in Fiji, associated with the high incidence and prevalence of GAS pharyngitis and RF in children [42, 43]. Meanwhile, the heavy economic burden caused by RHD strained local medical resources [44]. RHD remained a common cause of morbidity and mortality of acquired heart disease among children and young men in low and middle-SDI areas; thus, RHD control was regarded as an unfinished global agenda [45].

## Conclusions

Pronounced decreasing trends of death and DALYs due to RHD were observed worldwide from 1990 to 2019, indicating progress in the current management and treatment of RHD. However, the increasing trends of incidence and prevalence indicated that RHD remains a substantial challenge globally, and more effective strategies are needed for the prevention and control of the disease.

## Supplementary Information

**Additional file 1: Figure S1**. The number of RHD prevalence in age groups, SDI areas and geographic regions from 1990 to 2019. (A), the prevalence number in age groups; (B) the prevalence number in SDI areas; (C) the prevalence number in geographical regions. RHD: rheumatic heart disease; SDI, sociodemographic index. **Figure S2**. The ASR, percentage changes, and EAPCs of RHD prevalence at the national level, 1990-2019. (A), the ASR in 2019; (B), the percentage changes in number between 2000 and 2019; (C), the EAPCs in countries/territories, respectively. Countries/territories with an extreme value were annotated. RHD: rheumatic heart disease; ASR, age- standardized rate; EAPC, estimated annual percentage change. **Figure S3**. The death number of RHD in age groups, SDI areas, and geographic regions from 1990 to 2019. (A), (B), and (C) were the death number in age groups, SDI areas, and geographical regions, respectively. RHD: rheumatic heart disease; SDI, sociodemographic index. **Figure S4**. The ASR, percentage changes, and EAPCs of death caused by RHD at the national level, 1990-2019. (A), the ASR in 2019; (B), the percentage changes in number between 2000 and 2019; (C), the EAPCs in countries/territories, respectively. Countries/territories with an extreme value were annotated. RHD: rheumatic heart disease; ASR, age-standardized rate; EAPC, estimated annual percentage change. **Figure S5**. The number of DALYs caused by RHD in age groups, SDI areas, and geographic regions from 1990 to 2019. (A), (B), and (C) were the death number in age groups, SDI areas, and geographical regions, respectively. RHD: rheumatic heart disease; SDI, sociodemographic index. DALYs, disability-adjusted life years. **Figure S6**. The ASR, percentage changes, and EAPCs of DALYs caused by RHD at the national level, 1990-2019. (A), the ASR in 2019; (B), the percentage changes in number between 2000 and 2019; (C), the EAPCs in countries/territories, respectively. Countries/territories with an extreme value were annotated. RHD: rheumatic heart disease; ASR, age-standardized rate; EAPC, estimated annual percentage change; DALYs, disability-adjusted life years. **Figure S7**. The rate of death and DALYs caused by RHD by age and attributable risks from 1990 to 2019. (A) the attributable risks-related all age rate of death in age groups; (B) age-standardized rate of death from 1990 to 2019; (C) the attributable risks-related all age rate of DALYs in age groups; (D) age-standardized rate of DALYs from 1990 to 2019. In the (A) and (C), the upper column in each group is the data in 1990, and the lower column is in 2019. RHD: rheumatic heart disease; DALYs, disability-adjusted life years. **Figure S8**. The distribution of rate of death caused by RHD by sex, age and attributable risks. (A) the attributable risks-related all age rate of death in males, age groups; (B) age-standardized rate of death in males from 1990 to 2019; (C) the attributable risks-related all age rate of death in females, age groups; (D) age-standardized rate of death in females from 1990 to 2019. In the (A) and (C), the upper column in each group is the data in 1990, and the lower column is in 2019. RHD: rheumatic heart disease. **Figure S9**. The distribution of rate of DALYs caused by RHD by sex, age and attributable risks. (A) the attributable risks-related all age rate of DALYs in males, age groups; (B) age-standardized rate of DALYs in males from 1990 to 2019; (C) the attributable risks-related all age rate of DALYs in females, age groups; (D) age-standardized rate of DALYs in females from 1990 to 2019. In the (A) and (C), the upper column in each group is the data in 1990, and the lower column is in 2019. RHD: rheumatic heart disease; DALYs, disability-adjusted life years. **Table S1**. The number of RHD in 2019, and the percentage changes in number during the period 1990-2019 in age groups. **Table S2**. The age-standardized rate of incidence and prevalence of RHD at national level and both sexes in 2019, and percentage changes and EAPCs from 1990 to 2019. **Table S3**. The age-standardized rate of death and DALYs caused by RHD at national level and both sexes in 2019, and percentage changes and EAPCs from 1990 to 2019. **Table S4**. The age-standardized rate of death and DALYs caused by RHD at national level and both sexes in 2019, and percentage changes and EAPCs from 1990 to 2019. **Table S5**. The number and age-standardized rate of death and DALYs due to RHD in attributable risk factors globally, in sexes, in 2019, and percentage change in number and the EAPCs from 1990 to 2019. **Table S6**. The EAPCs of death and DALYs due to RHD in attributable risk factors in SDI quintiles from 1990 to 2019.

## Data Availability

All data during this study are included in this published article and its supplementary information files.

## Abbreviations

RHD: Rheumatic heart disease
ARF: Acute rheumatic fever
GAS: Group A streptococcus
GBD: Global Burden of Disease
DALYs: Disability-adjusted life years
ASR: Age-standardized rate
UI: Uncertainty interval
CI: Confidence interval
EAPC: Estimated annual percentage change
GHDx: Global Health Data Exchange
SDI: Socio-Demographic Index
HDI: Human Development Index
WHO: World Health Organization
WHF: World Heart Federation
