# Supplementary material for: Global burden of rheumatic heart disease: trends from 1990 to 2019
Source: Arthritis Res Ther. 2022 Jun 11;24:138. doi: 10.1186/s13075-022-02829-3 (PMC9188068; doi:10.1186/s13075-022-02829-3)
Supplement: Supplementary file 1 — Additional file 1: Figure S1. The number of RHD prevalence in age groups, SDI areas and geographic regions from 1990 to 2019. (A), the prevalence number in age groups; (B) the prevalence number in SDI areas; (C) the prevalence number in geographical regions. RHD: rheumatic heart disease; SDI, sociodemographic index. Figure S2. The ASR, percentage changes, and EAPCs of RHD prevalence at the national level, 1990-2019. (A), the ASR in 2019; (B), the percentage changes in number between 2000 and 2019; (C), the EAPCs in countries/territories, respectively. Countries/territories with an extreme value were annotated. RHD: rheumatic heart disease; ASR, age- standardized rate; EAPC, estimated annual percentage change. Figure S3. The death number of RHD in age groups, SDI areas, and geographic regions from 1990 to 2019. (A), (B), and (C) were the death number in age groups, SDI areas, and geographical regions, respectively. RHD: rheumatic heart disease; SDI, sociodemographic index. Figure S4. The ASR, percentage changes, and EAPCs of death caused by RHD at the national level, 1990-2019. (A), the ASR in 2019; (B), the percentage changes in number between 2000 and 2019; (C), the EAPCs in countries/territories, respectively. Countries/territories with an extreme value were annotated. RHD: rheumatic heart disease; ASR, age-standardized rate; EAPC, estimated annual percentage change. Figure S5. The number of DALYs caused by RHD in age groups, SDI areas, and geographic regions from 1990 to 2019. (A), (B), and (C) were the death number in age groups, SDI areas, and geographical regions, respectively. RHD: rheumatic heart disease; SDI, sociodemographic index. DALYs, disability-adjusted life years. Figure S6. The ASR, percentage changes, and EAPCs of DALYs caused by RHD at the national level, 1990-2019. (A), the ASR in 2019; (B), the percentage changes in number between 2000 and 2019; (C), the EAPCs in countries/territories, respectively. Countries/territories with an extreme value we [file 13075_2022_2829_MOESM1_ESM.docx]

**Supplementary figure 1**. The number of RHD prevalence in age groups, SDI areas and geographic regions from 1990 to 2019. (A), the prevalence number in age groups; (B) the prevalence number in SDI areas; (C) the prevalence number in geographical regions. RHD: rheumatic heart disease; SDI, sociodemographic index.

**
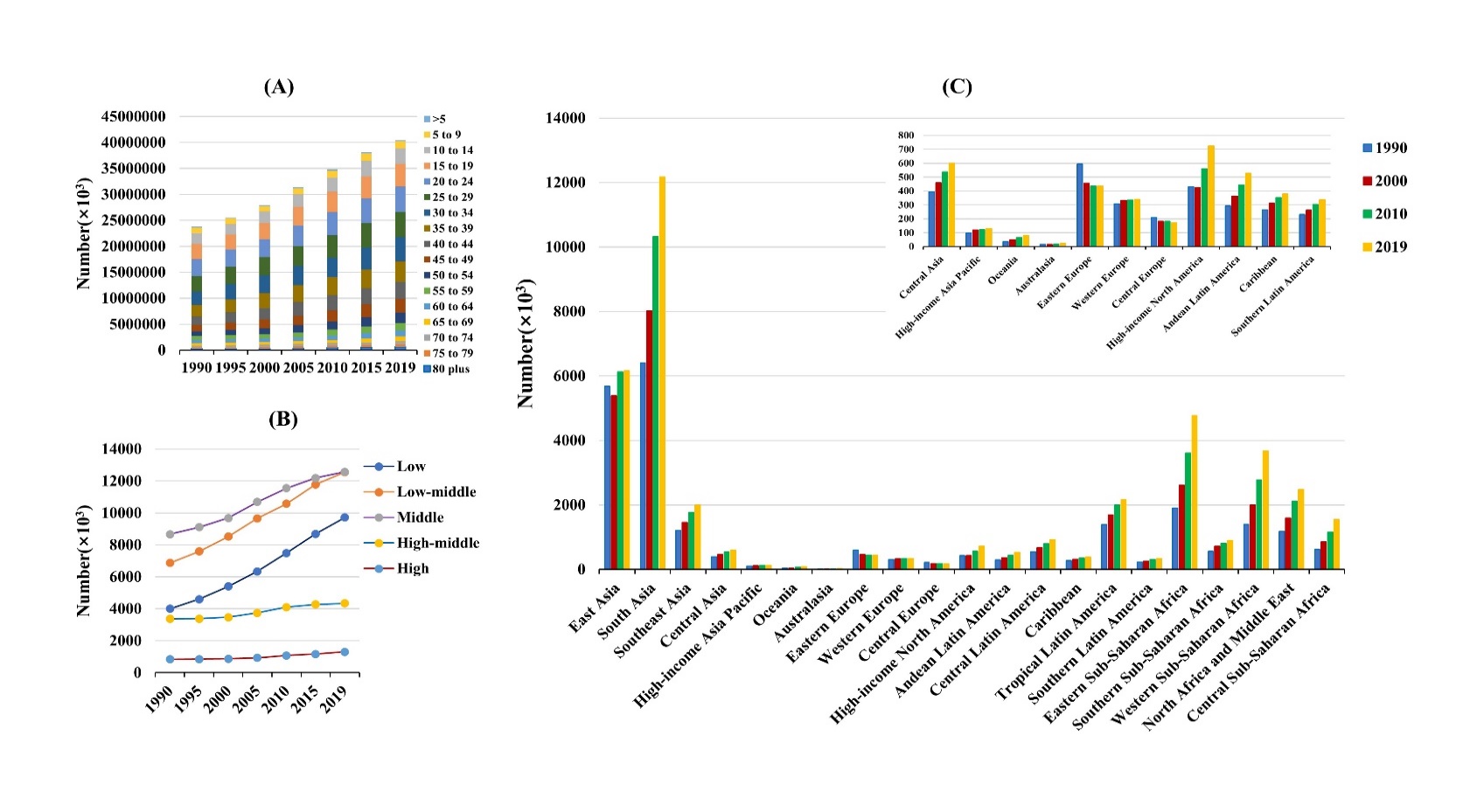
**

**Supplementary figure 2**. The ASR, percentage changes, and EAPCs of RHD prevalence at the national level, 1990-2019. (A), the ASR in 2019; (B), the percentage changes in number between 2000 and 2019; (C), the EAPCs in countries/territories, respectively. Countries/territories with an extreme value were annotated. RHD: rheumatic heart disease; ASR, age- standardized rate; EAPC, estimated annual percentage change.

**
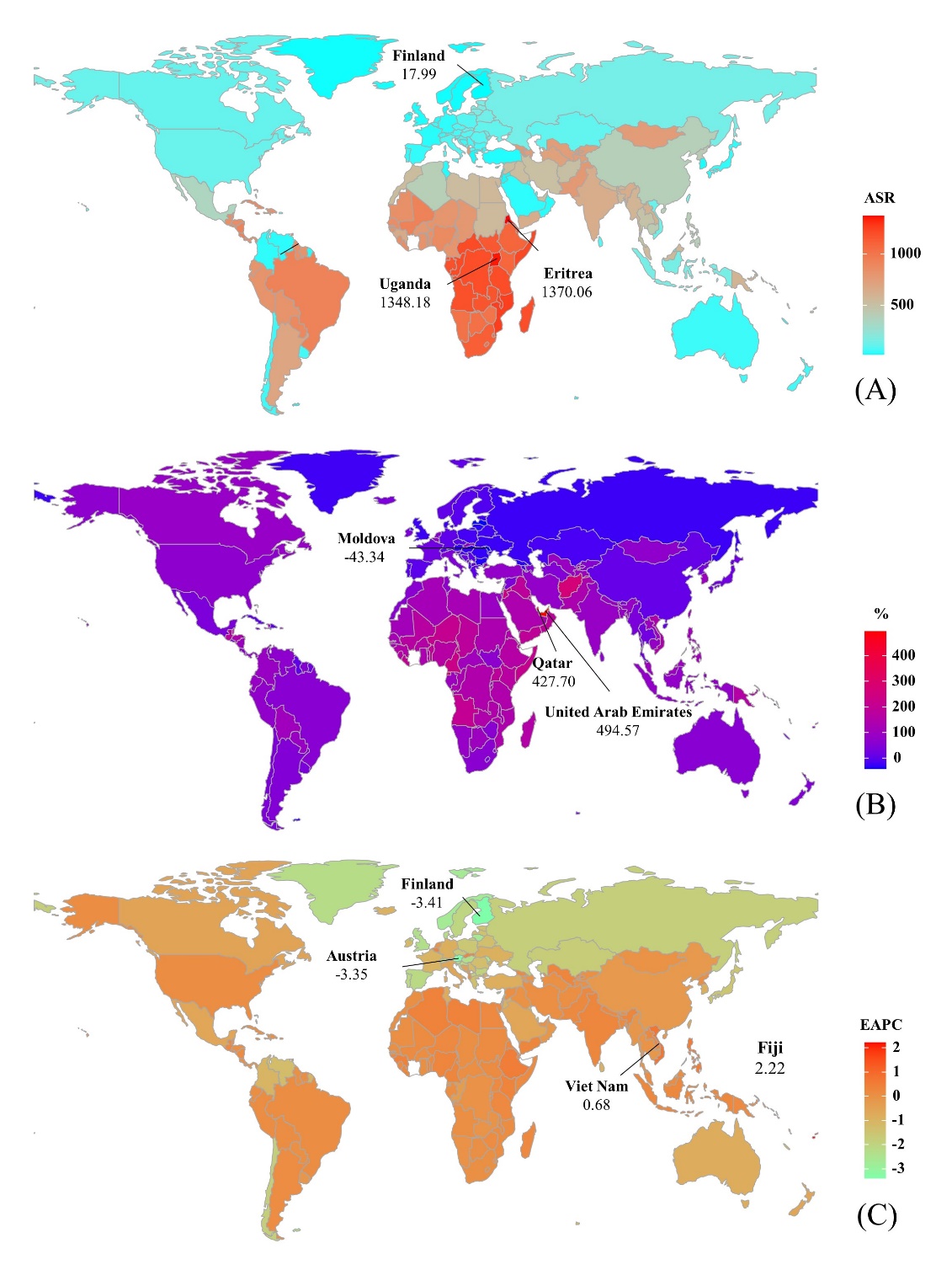
**

**Supplementary figure 3**. The death number of RHD in age groups, SDI areas, and geographic regions from 1990 to 2019. (A), (B), and (C) were the death number in age groups, SDI areas, and geographical regions, respectively. RHD: rheumatic heart disease; SDI, sociodemographic index.

**
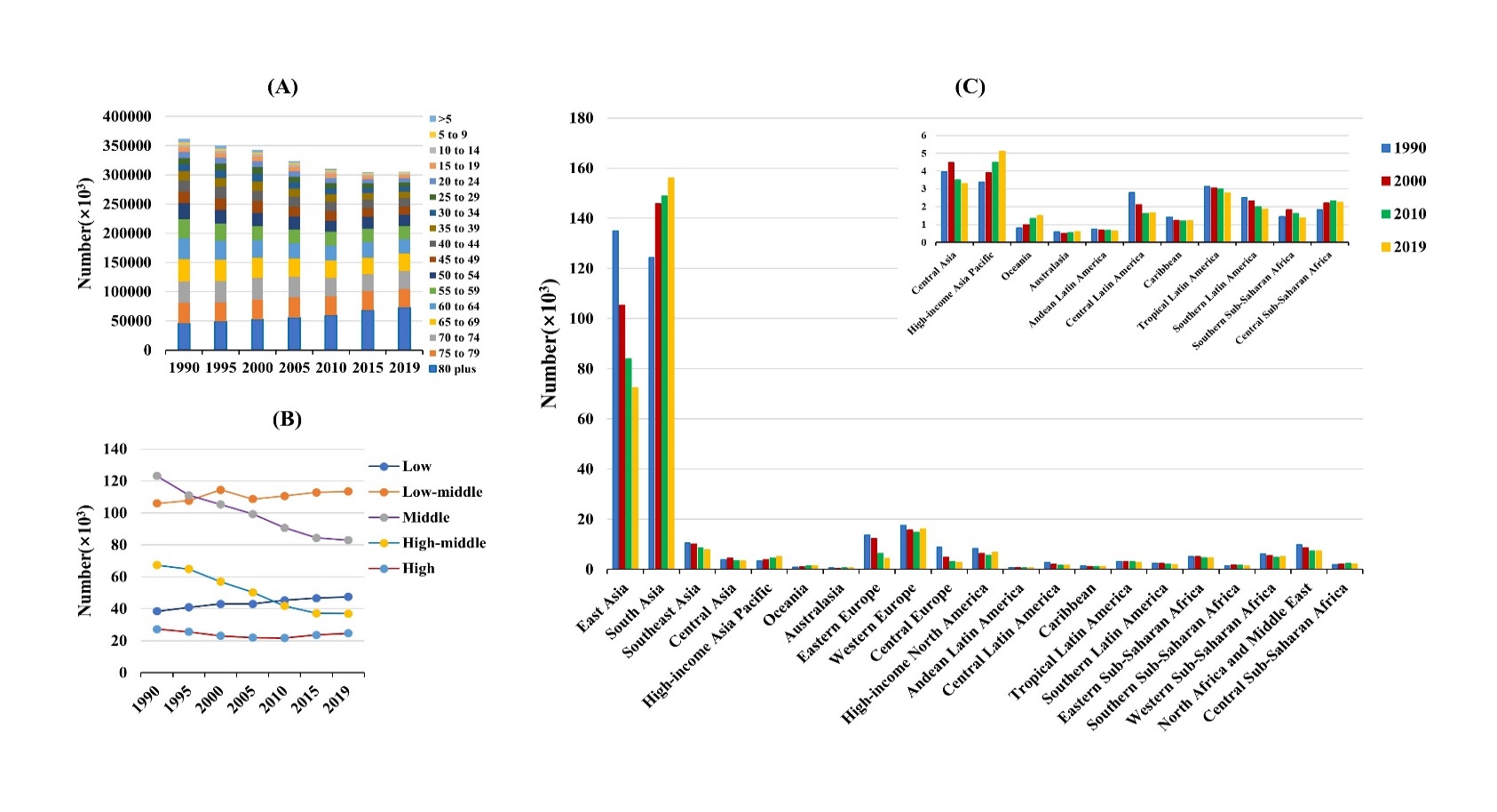
**

**Supplementary figure 4**. The ASR, percentage changes, and EAPCs of death caused by RHD at the national level, 1990-2019. (A), the ASR in 2019; (B), the percentage changes in number between 2000 and 2019; (C), the EAPCs in countries/territories, respectively. Countries/territories with an extreme value were annotated. RHD: rheumatic heart disease; ASR, age-standardized rate; EAPC, estimated annual percentage change.

**
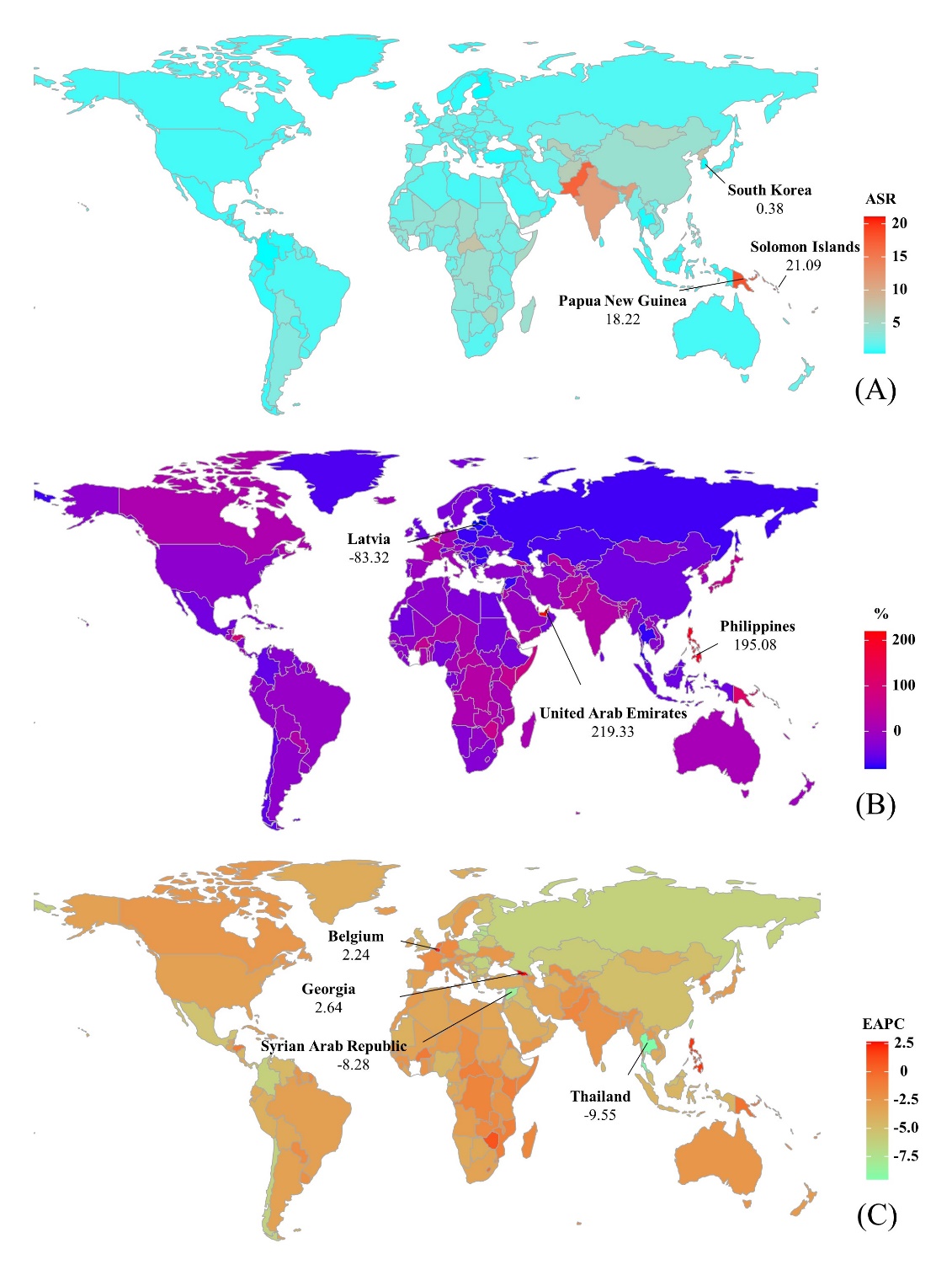
**

**Supplementary figure 5**. The number of DALYs caused by RHD in age groups, SDI areas, and geographic regions from 1990 to 2019. (A), (B), and (C) were the death number in age groups, SDI areas, and geographical regions, respectively. RHD: rheumatic heart disease; SDI, sociodemographic index. DALYs, disability-adjusted life years.

**
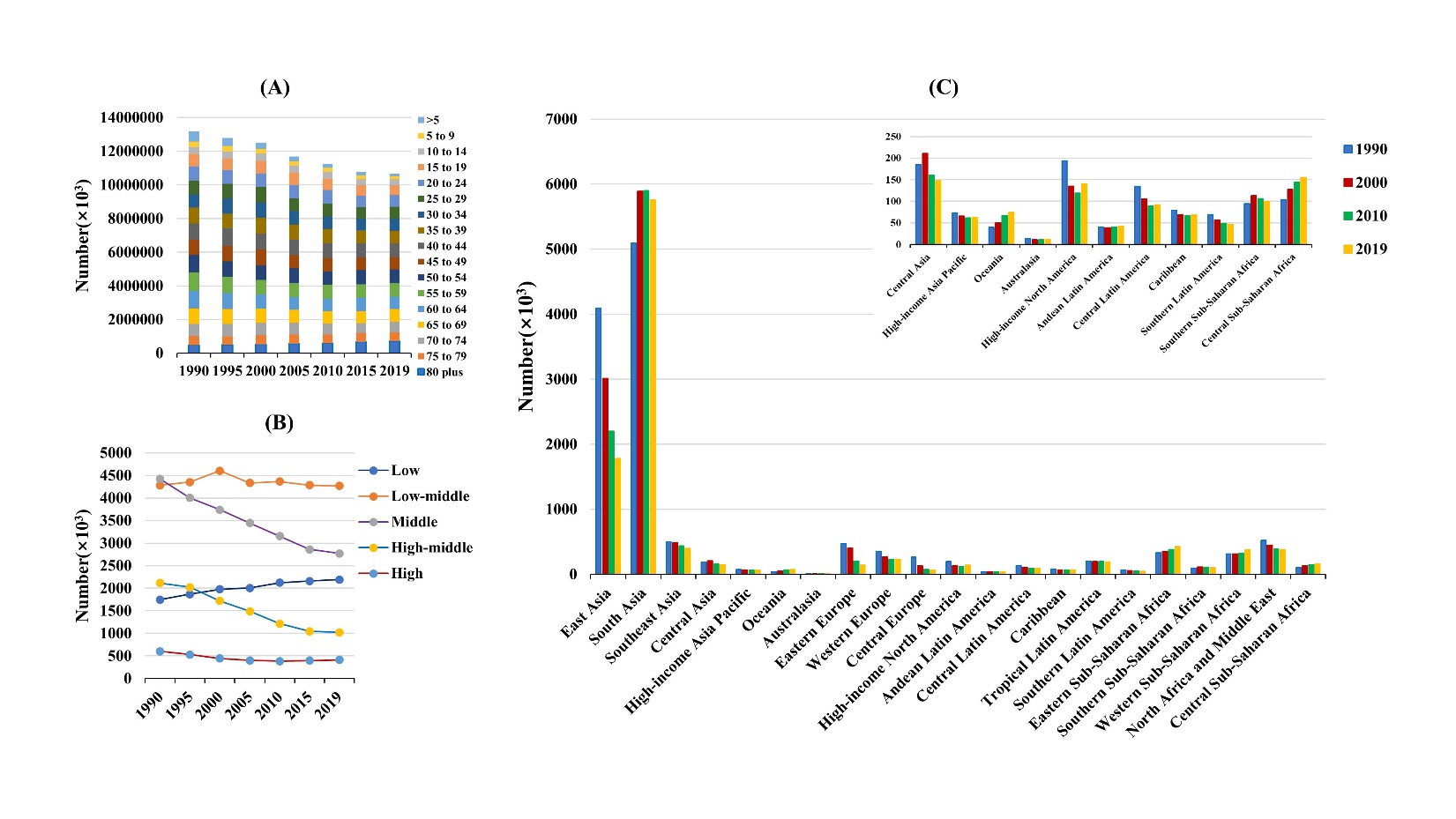
**

**Supplementary figure 6**. The ASR, percentage changes, and EAPCs of DALYs caused by RHD at the national level, 1990-2019. (A), the ASR in 2019; (B), the percentage changes in number between 2000 and 2019; (C), the EAPCs in countries/territories, respectively. Countries/territories with an extreme value were annotated. RHD: rheumatic heart disease; ASR, age-standardized rate; EAPC, estimated annual percentage change; DALYs, disability-adjusted life years.

**
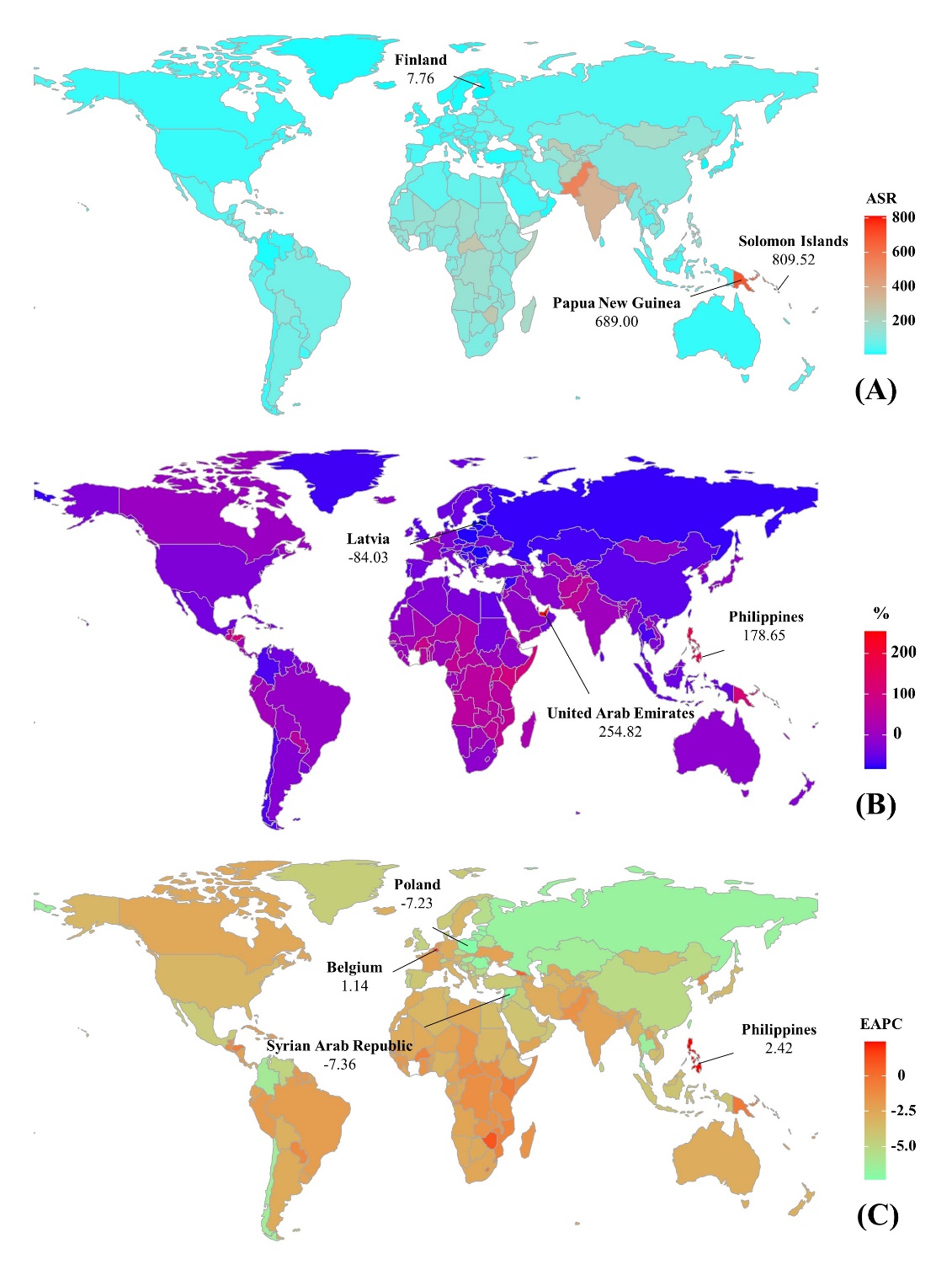
**

**Supplementary figure 7.** The rate of death and DALYs caused by RHD by age and attributable risks from 1990 to 2019. (A) the attributable risks-related all age rate of death in age groups; (B) age-standardized rate of death from 1990 to 2019; (C) the attributable risks-related all age rate of DALYs in age groups; (D) age-standardized rate of DALYs from 1990 to 2019. In the (A) and (C), the upper column in each group is the data in 1990, and the lower column is in 2019. RHD: rheumatic heart disease; DALYs, disability-adjusted life years.

**
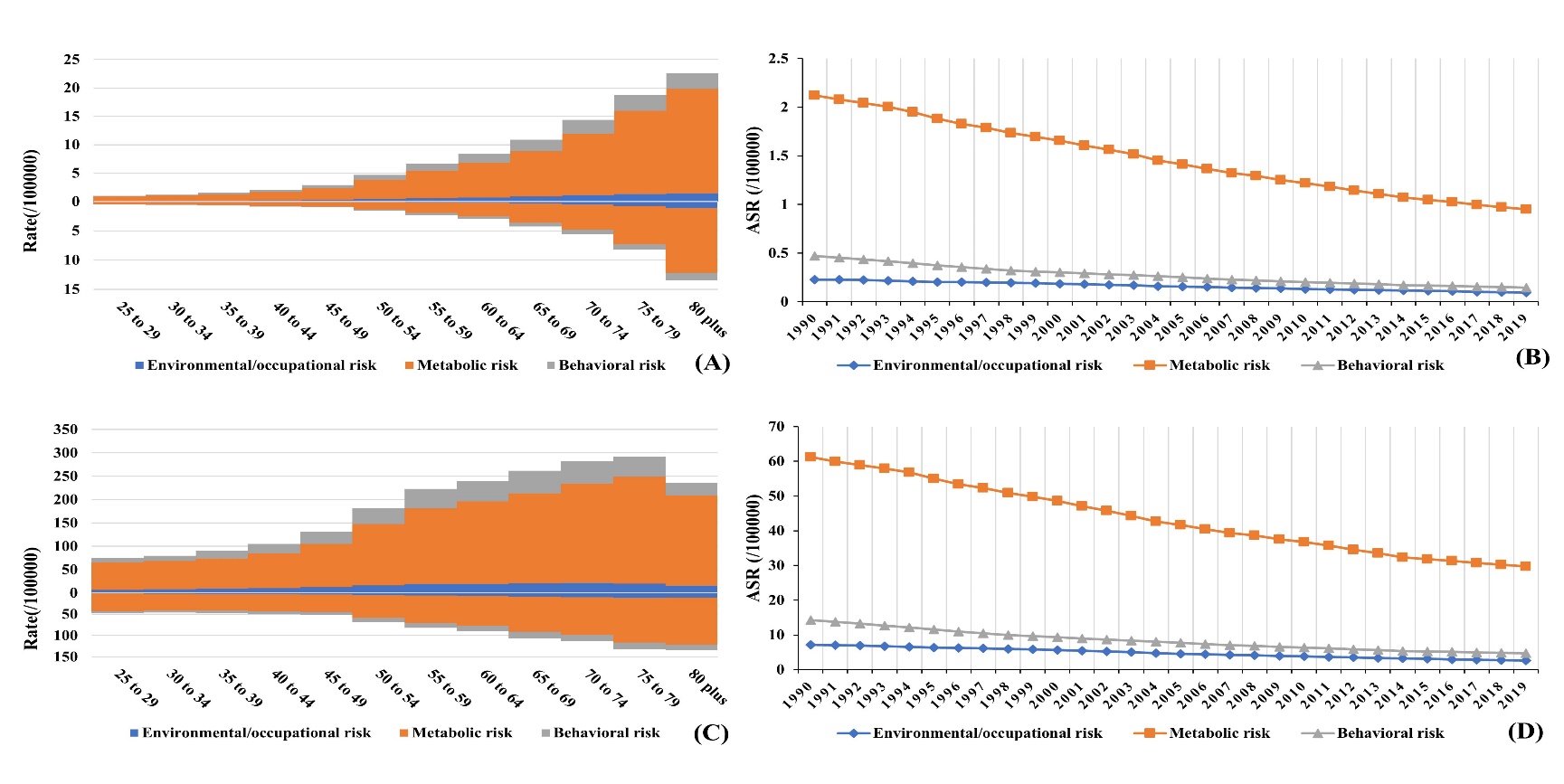
**

**Supplementary figure 8.** The distribution of rate of death caused by RHD by sex, age and attributable risks. (A) the attributable risks-related all age rate of death in males, age groups; (B) age-standardized rate of death in males from 1990 to 2019; (C) the attributable risks-related all age rate of death in females, age groups; (D) age-standardized rate of death in females from 1990 to 2019. In the (A) and (C), the upper column in each group is the data in 1990, and the lower column is in 2019. RHD: rheumatic heart disease.


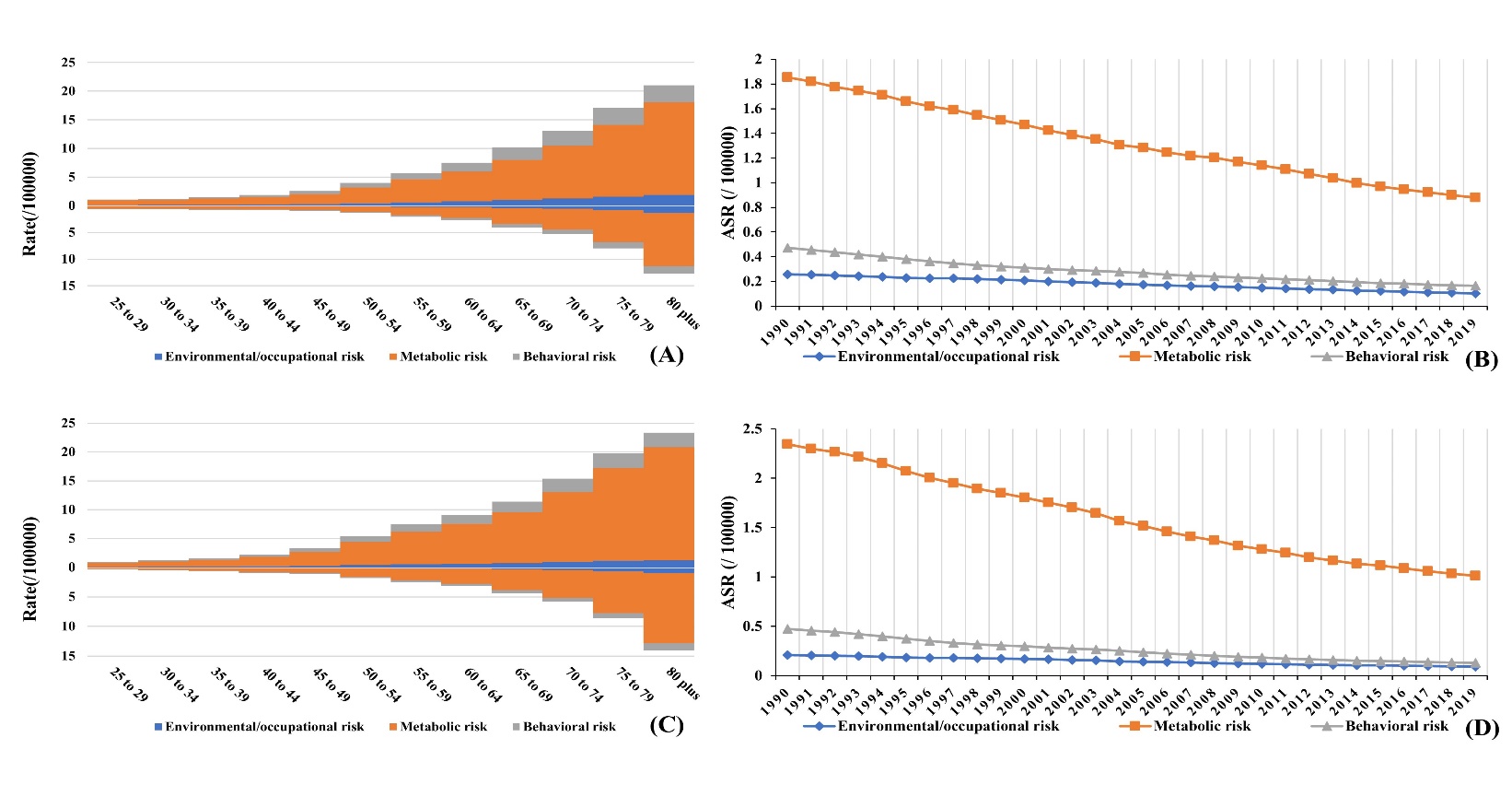


**Supplementary figure 9.** The distribution of rate of DALYs caused by RHD by sex, age and attributable risks. (A) the attributable risks-related all age rate of DALYs in males, age groups; (B) age-standardized rate of DALYs in males from 1990 to 2019; (C) the attributable risks-related all age rate of DALYs in females, age groups; (D) age-standardized rate of DALYs in females from 1990 to 2019. In the (A) and (C), the upper column in each group is the data in 1990, and the lower column is in 2019. RHD: rheumatic heart disease; DALYs, disability-adjusted life years.


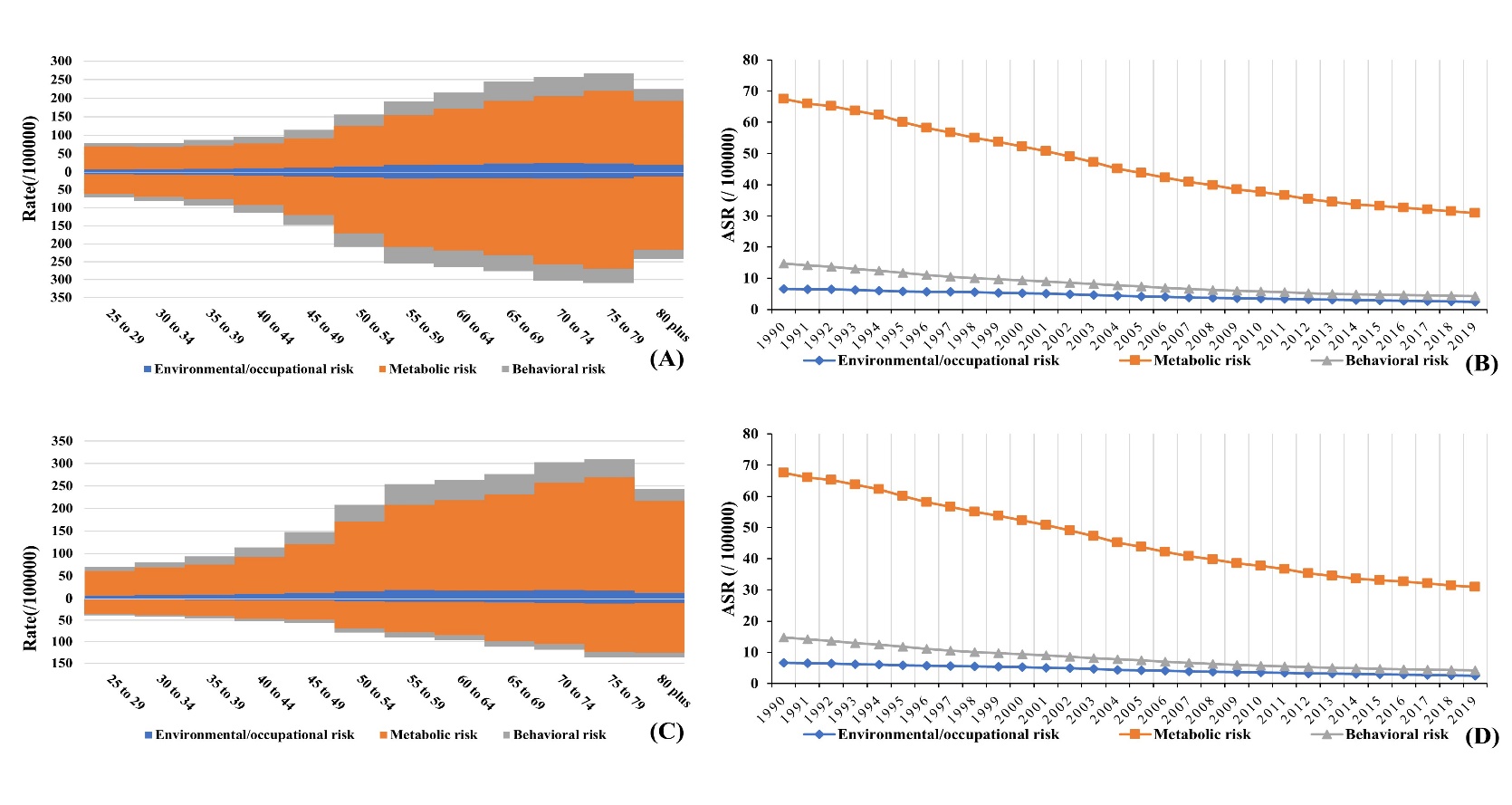


**Supplementary table 1**. The number of RHD in 2019, and the percentage changes in number during the period 1990-2019 in age groups.

| **Age Groups** | **Incidence** | | **Prevalence** | | **Death** | | **DALYs** | |
| --- | --- | --- | --- | --- | --- | --- | --- | --- |
|  | Number  ×10^3^(95% UI) | Percentage (%) | Number  ×10^3^(95% UI) | Percentage (%) | Number  ×10^3^(95% UI) | Percentage  (%) | Number  ×10^3^(95% UI) | Percentage (%) |
| **﹤5** | 146.40(76.85-240.72) | 23.26 | 241.15(127.06-394.34) | 22.72 | 1.66(1.24-2.18) | −75.35 | 154.27(117.70-200.20) | −73.74 |
| **5 to 9** | 382.95(195.03-655.61) | 37.35 | 1408.94(882.26-2107.26) | 33.71 | 1.44(1.15-1.82) | −58.05 | 186.29(145.27-244.14) | −43.95 |
| **10 to 14** | 508.66(275.54-840.17) | 52.31 | 2992.69(1767.81-4735.98) | 46.31 | 2.79(2.31-3.26) | −34.34 | 357.32(276.53-475.59) | −15.56 |
| **15 to 19** | 483.19(254.21-729.90) | 51.73 | 4329.45(2747.14-6533.43) | 47.10 | 5.42(4.75-6.19) | −34.33 | 595.10(478.51-752.33) | −18.61 |
| **20 to 24** | 372.18(196.54-568.28) | 50.62 | 4860.34(3454.47-6559.12) | 49.49 | 6.78(5.90-7.77) | −34.38 | 684.02(563.77-842.78) | −18.90 |
| **25 to 29** | 281.85(171.83-403.30) | 60.94 | 4910.47(3484.43-6513.77) | 63.41 | 7.59(6.57-8.64) | −29.92 | 702.29(578.70-847.99) | −13.40 |
| **30 to 34** | 207.21(140.09-278.84) | 76.55 | 4674.78(3450.75-6193.44) | 84.44 | 8.75(7.18-10.10) | −25.95 | 718.60(592.37-860.34) | −9.03 |
| **35 to 39** | 133.09(87.47-191.81) | 62.85 | 3953.26(3052.12-5089.81) | 77.62 | 11.01(8.64-12.97) | −31.53 | 757.72(617.39-903.14) | −19.15 |
| **40 to 44** | 76.34(38.68-125.62) | 65.88 | 3241.63(2555.92-4123.29) | 98.16 | 13.99(10.73-16.68) | −27.26 | 810.37(648.30-960.32) | −17.16 |
| **45 to 49** | 34.00(20.94-49.55) | 47.64 | 2654.47(2108.9-3317.59) | 120.6 | 14.22(11.02-16.96) | −27.51 | 726.31(580.64-854.94) | −17.52 |
| **50 to 54** | 18.19(13.53-23.50) | −11.44 | 2022.89(1610.39-2537.06) | 113.33 | 19.60(14.53-23.27) | −27.63 | 830.21(628.71-978.08) | −21.13 |
| **55 to 59** | 23.56(14.78-33.39) | 11.69 | 1458.96(1173.81-1830.23) | 95.66 | 22.47(17.49-26.25) | −31.80 | 803.49(633.02-933.63) | −27.37 |
| **60 to 64** | 25.32(17.65-33.42) | 17.22 | 1122.37(918.65-1384.23) | 77.78 | 24.26(19.97-27.79) | −32.53 | 734.90(611.28-829.32) | −28.92 |
| **65 to 69** | 24.30(16.77-32.30) | 35.55 | 889.83(742.99-1061.74) | 86.82 | 30.10(25.60-34.08) | −20.83 | 755.86(645.90-849.22) | −17.47 |
| **70 to 74** | 21.46(15.07-28.73) | 59.94 | 642.67(548.31-747.99) | 94.25 | 30.71(26.28-34.27) | −15.51 | 625.65(538.09-694.31) | −12.40 |
| **75 to 79** | 17.13(11.25-23.78) | 47.10 | 459.59(396.20-525.1) | 74.13 | 31.65(27.20-35.24) | −10.25 | 504.80(435.24-559.53) | −8.07 |
| **＞80** | 33.63(26.8-41.88) | 101.08 | 638.85(559.97-731.12) | 140.36 | 73.22(61.17-81.35) | 59.22 | 726.67(618.56-803.56) | 50.83 |

RHD: rheumatic heart disease; DALYs, disability-adjusted life years.

**Supplementary table 2**. The age-standardized rate of incidence and prevalence of RHD at national level and both sexes in 2019, and percentage changes and EAPCs from 1990 to 2019

|  | **Incidence** | | | **Prevalence** | | |
| --- | --- | --- | --- | --- | --- | --- |
| **Characteristics** | ASR/100,000  (95% UI) | Percentage (%) | EAPC  (95%CI) | ASR/100,000  (95% UI) | Percentage (%) | EAPC  (95%CI) |
| Afghanistan | 36.95(28.5-46.45) | 276.82 | 0.03(0-0.07) | 504.92(394.25-627.93) | 266.41 | 0.05(0.03-0.06) |
| Albania | 40.73(30.59-51.95) | −46.25 | −0.14(−0.15-−0.12) | 759.08(588.45-953.56) | −15.36 | 0.03(0.01-0.06) |
| Algeria | 24.15(18.43-30.78) | 57.26 | 0.33(0.22-0.44) | 400.06(312.56-500.78) | 115.96 | 0.37(0.27-0.47) |
| American Samoa | 38.99(29.84-49.83) | −0.29 | 0.14(0.13-0.14) | 688.49(542.36-872.52) | 21.14 | 0.1(0.09-0.1) |
| Andorra | 2.42(2.05-2.81) | 69.44 | −1.52(−1.66-−1.38) | 29.84(24.52-35.47) | 76.7 | −1.13(−1.24-−1.02) |
| Angola | 77.76(57.3-99.5) | 204.3 | −0.02(−0.05-0.01) | 1187.01(913.65-1502.01) | 201.8 | 0.06(0.05-0.08) |
| Antigua and Barbuda | 45.8(34.44-58.62) | 13.44 | −0.07(−0.08-−0.05) | 866.74(679.87-1091.19) | 56.66 | −0.01(−0.02-0) |
| Argentina | 38(28.73-47.96) | 23.61 | 0.01(−0.01-0.03) | 693.5(541.84-862.62) | 46.28 | 0.12(0.09-0.14) |
| Armenia | 42.52(31.84-53.96) | −35.71 | −0.33(−0.39-−0.27) | 797.21(617.34-999.09) | −13.2 | −0.19(−0.22-−0.16) |
| Australia | 3.63(3.18-4.17) | 46.91 | −1.19(−1.4-−0.98) | 49.67(41.64-58.26) | 56.39 | −0.79(−0.95-−0.63) |
| Austria | 5.00(4.50-5.55) | −14.41 | −2.72(−2.95-−2.49) | 58.22(51.24-65.6) | −28.8 | −3.35(−3.65-−3.06) |
| Azerbaijan | 41.86(31.87-53.1) | 12.46 | −0.21(−0.28-−0.14) | 734.34(573.64-915.93) | 49.82 | −0.08(−0.1-−0.07) |
| Bahamas | 48.62(36.31-61.74) | 16.81 | 0.01(−0.01-0.02) | 839.59(647.55-1059.21) | 50.41 | 0.02(0.01-0.03) |
| Bahrain | 3.06(2.65-3.52) | 237.80 | −1.37(−1.49-−1.24) | 36.22(30.04-43.05) | 256.66 | −1.04(−1.14-−0.93) |
| Bangladesh | 36.3(26.97-46.18) | 37.69 | −0.02(−0.04-0) | 562.24(431.86-706.05) | 83.1 | 0.13(0.1-0.16) |
| Barbados | 47.56(35.72-60.45) | −12.48 | 0.01(−0.02-0.04) | 868.52(673.92-1089.54) | 16.85 | 0.02(0.01-0.04) |
| Belarus | 7.97(6.83-9.33) | −36.89 | −2.10(−2.20-−2.00) | 134.71(112.79-161.46) | −23.02 | −1.34(−1.54-−1.14) |
| Belgium | 4.47(3.91-5.13) | 120.58 | 1.08(0.76-1.39) | 44.49(36.78-53.47) | 78.41 | 0.38(−0.11-0.88) |
| Belize | 50.23(37.46-63.69) | 95.61 | −0.03(−0.05-−0.01) | 836.74(649.13-1044.61) | 152.77 | 0.05(0.04-0.06) |
| Benin | 54.77(41.5-69.34) | 175.13 | 0.13(0.11-0.15) | 813.14(629.15-1023.4) | 191.22 | 0.15(0.13-0.16) |
| Bermuda | 2.76(2.29-3.27) | 30.4 | −1.28(−1.37-−1.2) | 40.25(33.35-48.14) | 31.58 | −1.19(−1.28-−1.1) |
| Bhutan | 36.68(28.06-46.73) | 9.32 | −0.11(−0.13-−0.09) | 580.88(452.34-729.74) | 45.84 | 0.01(0-0.03) |
| Bolivia  (Plurinational State of) | 51.37(38.92-65.21) | 68.07 | 0.02(0-0.03) | 810.88(627.12-1013.14) | 103.78 | 0.09(0.08-0.11) |
| Bosnia and Herzegovina | 3.4(2.9-4) | −29.48 | −1.57(−1.81-−1.34) | 47.55(39.35-56.81) | 9.87 | −0.47(−0.66-−0.27) |
| Botswana | 69.13(51.23-88.44) | 53.13 | −0.05(−0.06-−0.03) | 1029(797.11-1304.05) | 99 | 0.02(0.01-0.03) |
| Brazil | 54.2(40.73-68.51) | 13.54 | 0.02(0-0.03) | 918.52(716.01-1142.45) | 55.41 | 0.08(0.07-0.09) |
| Brunei Darussalam | 5.43(4.76-6.22) | 114.29 | −1.12(−1.24-−1.01) | 72.66(60.4-87.4) | 112.53 | −0.99(−1.08-−0.89) |
| Bulgaria | 4.62(4.08-5.23) | −54.77 | −2.96(−3.24-−2.67) | 61.14(52.04-71.98) | −36.67 | −1.91(−2.19-−1.62) |
| Burkina Faso | 54.84(41.27-70.49) | 142.92 | 0.03(0.02-0.04) | 778.99(600.89-986.1) | 156.37 | 0.07(0.06-0.07) |
| Burundi | 84.8(64.18-108.95) | 130.88 | 0.16(0.15-0.17) | 1183.52(908.15-1506.13) | 132.83 | 0.12(0.11-0.12) |
| Cabo Verde | 50.36(37.49-64.94) | 36.91 | −0.03(−0.04-−0.02) | 887.17(687.02-1131.61) | 90.1 | 0.06(0.05-0.07) |
| Cambodia | 30.04(23.12-37.91) | 41.35 | −0.26(−0.39-−0.13) | 429.21(336.72-542.44) | 80.7 | −0.18(−0.32-−0.05) |
| Cameroon | 55.55(41.53-71.47) | 210.18 | 0.28(0.24-0.31) | 803.08(620.35-1018.58) | 227.36 | 0.28(0.24-0.31) |
| Canada | 8.6(7.17-10.11) | 74.88 | −0.63(−0.7-−0.56) | 111.72(94.17-130.31) | 81.44 | −0.54(−0.6-−0.47) |
| Central African Republic | 88.44(66.49-112.67) | 103.99 | 0.1(0.09-0.12) | 1205.39(916.82-1545.24) | 107.13 | 0.06(0.04-0.07) |
| Chad | 54.47(41.21-69.3) | 189.63 | 0.01(0-0.01) | 781.43(604.72-988.2) | 179.82 | 0.05(0.05-0.06) |
| Chile | 3.84(3.3-4.47) | 6.44 | −2.72(−2.86-−2.58) | 54.26(45.23-63.96) | 39.87 | −1.81(−1.95-−1.68) |
| China | 23.95(18.62-29.89) | −27.53 | −0.5(−0.68-−0.33) | 390.24(310.29-481.99) | 7.64 | −0.23(−0.41-−0.05) |
| Colombia | 2.59(2.18-3.03) | 52.95 | −1.79(−2.06-−1.52) | 36.52(30.33-43.74) | 86.99 | −1.03(−1.28-−0.78) |
| Comoros | 78.1(58.34-101.49) | 44.46 | 0.13(0.12-0.13) | 1219.49(937.98-1554.81) | 78.23 | 0.09(0.08-0.1) |
| Congo | 81.36(60.94-103.81) | 104.15 | −0.3(−0.45-−0.16) | 1206.48(934.32-1528.12) | 130.62 | −0.32(−0.46-−0.17) |
| Cook Islands | 8.97(7.86-10.18) | 15.47 | −0.73(−0.85-−0.61) | 151.55(130.55-174.08) | 46.90 | −0.21(−0.33-−0.09) |
| Costa Rica | 47.51(35.94-60.13) | 17.63 | −0.11(−0.12-−0.1) | 918.66(718.26-1147.57) | 67.01 | 0(−0.01-0.01) |
| Croatia | 4.17(3.74-4.63) | −32.53 | −2.35(−2.75-−1.94) | 52.88(45.13-61.42) | −17.2 | −2.04(−2.34-−1.74) |
| Cuba | 47.32(35.29-59.92) | −26.41 | −0.03(−0.06-0.01) | 870.92(679.29-1096.17) | 1.79 | 0.02(0-0.03) |
| Cyprus | 4.53(4.01-5.13) | 27.55 | −2.74(−3.01-−2.46) | 32.88(26.95-40.21) | 39.41 | −2.24(−2.54-−1.95) |
| Czechia | 5.07(4.43-5.84) | −22.62 | −2.17(−2.56-−1.79) | 65.69(53.62-79.97) | −5.91 | −1.65(−1.99-−1.31) |
| Cote d'Ivoire | 56.55(42.6-71.87) | 114.17 | 0.12(0.09-0.16) | 811.5(631.08-1027.69) | 132.82 | 0.09(0.07-0.12) |
| Democratic People's Republic of Korea | 28.18(21.51-35.78) | 8.01 | 0.09(0.07-0.11) | 425.6(335.06-541.24) | 33.77 | 0.02(0.01-0.04) |
| Democratic Republic of  the Congo | 82.97(61.85-105.87) | 137.94 | 0.06(0.02-0.1) | 1198.49(912.02-1513.56) | 144.66 | −0.03(−0.05-−0.01) |
| Denmark | 2.02(1.75-2.32) | −32.78 | −2.81(−3.71-−1.9) | 20.99(16.96-25.39) | −24.12 | −2.27(−3.01-−1.53) |
| Djibouti | 76.17(56.83-98.14) | 128.41 | 0.21(0.19-0.23) | 1200.08(929.13-1516.34) | 181.36 | 0.21(0.2-0.22) |
| Dominica | 51.14(38.27-65.32) | −26.47 | −0.05(−0.06-−0.03) | 861.71(674.08-1075.14) | −1.07 | 0(−0.01-0.01) |
| Dominican Republic | 48.26(36.21-61) | 24.92 | −0.1(−0.1-−0.09) | 819.46(638.36-1027.47) | 59.52 | 0.02(0.02-0.03) |
| Ecuador | 47.85(35.91-60.97) | 50.77 | −0.02(−0.03-0) | 806.96(625.17-1005.82) | 86.7 | 0.05(0.03-0.07) |
| Egypt | 33.29(25.47-41.43) | 65.03 | 0.11(0-0.22) | 563.17(434.69-703.24) | 90.91 | 0.23(0.11-0.34) |
| El Salvador | 51.41(38.66-65.46) | 0.49 | −0.03(−0.04-−0.02) | 853.21(671.37-1078.86) | 30.77 | 0.09(0.08-0.1) |
| Equatorial Guinea | 71.42(53.39-91.85) | 215.05 | −0.69(−0.79-−0.6) | 1149.22(887.8-1446.94) | 266.26 | −0.24(−0.28-−0.21) |
| Eritrea | 93.56(70.93-119.54) | 120.88 | 0.25(0-0.49) | 1370.06(1054.58-1735.74) | 145.13 | 0.25(0-0.49) |
| Estonia | 8.28(6.91-9.89) | −44.06 | −2.05(−2.16-−1.95) | 152.6(125.59-183.87) | −23.94 | −1.4(−1.54-−1.27) |
| Eswatini | 70.72(53.08-90.85) | 33.00 | 0.01(0-0.02) | 1016.05(788.37-1289.87) | 59.16 | 0.04(0.03-0.04) |
| Ethiopia | 74.74(55.86-95.6) | 143.93 | 0.46(0.39-0.53) | 1084.02(833.96-1372.06) | 158.24 | 0.49(0.43-0.56) |
| Fiji | 30.29(23.14-39.16) | 52.54 | 2.17(1.48-2.86) | 480.06(378.47-613.63) | 83.22 | 2.22(1.53-2.91) |
| Finland | 1.48(1.25-1.73) | −30.71 | −3.87(−4.18-−3.56) | 17.99(14.73-21.67) | −10.87 | −3.41(−3.86-−2.96) |
| France | 3.42(3.01-3.88) | 23.61 | −1.23(−1.37-−1.08) | 33.13(27.3-39.56) | 21.21 | −0.99(−1.08-−0.9) |
| Gabon | 72.38(53.3-92.91) | 72.58 | 0.1(0.09-0.11) | 1180.44(900.41-1500.2) | 98.38 | 0.05(0.03-0.06) |
| Gambia | 57.54(43.05-73.3) | 141.64 | 0.22(0.21-0.24) | 860.32(661.24-1088.85) | 157.41 | 0.22(0.20-0.23) |
| Georgia | 43.51(33.32-55.01) | −44.65 | 0.09(0.05-0.13) | 768.8(597.27-959.89) | −34.46 | 0.14(0.11-0.16) |
| Germany | 4.23(3.78-4.75) | 13.42 | −0.95(−1.08-−0.83) | 36.85(30.7-44.26) | 1.70 | −0.86(−1.03-−0.69) |
| Ghana | 53.75(40.5-69.03) | 101.34 | −0.02(−0.05-0) | 809.73(628.73-1028.82) | 132.24 | 0.03(0.01-0.06) |
| Greece | 1.89(1.62-2.18) | −15.72 | −2.13(−2.49-−1.77) | 22.23(18.37-26.77) | −0.52 | −1.37(−1.60-−1.14) |
| Greenland | 11.24(9.31-13.28) | 23.49 | −1.88(−1.95-−1.8) | 142.97(118.63-168.57) | 40.39 | −1.29(−1.34-−1.23) |
| Grenada | 49.55(36.61-63.13) | −6.64 | −0.11(−0.13-−0.1) | 863.94(669.95-1089.07) | 34.83 | −0.01(−0.02-0) |
| Guam | 33.86(25.95-44.31) | 10.3 | 0.15(0.13-0.17) | 654.89(516.31-828.75) | 27.33 | 0.18(0.16-0.2) |
| Guatemala | 54.21(40.81-68.86) | 124 | 0.14(0.11-0.17) | 838.16(649.99-1056.08) | 168.56 | 0.23(0.2-0.26) |
| Guinea | 46.69(35.28-59.56) | 156.63 | 0.38(0.3-0.45) | 657.76(514.55-831.36) | 152.11 | 0.37(0.3-0.45) |
| Guinea-Bissau | 59.09(44.83-74.72) | 102.28 | 0.21(0.2-0.23) | 824.61(638.61-1037.24) | 116.47 | 0.21(0.19-0.22) |
| Guyana | 54.86(41.26-69.38) | −15.82 | −0.1(−0.11-−0.09) | 839.39(651.76-1049.68) | 3.37 | 0(−0.01-0.01) |
| Haiti | 59.55(45.34-76.18) | 95.57 | 0.08(0.07-0.09) | 828.62(639.15-1038.83) | 118.94 | 0.09(0.09-0.1) |
| Honduras | 53.98(40.71-68.24) | 93.61 | −0.02(−0.03-0) | 847.75(660.49-1062.93) | 137.57 | 0.04(0.03-0.06) |
| Hungary | 5.84(5.03-6.73) | −45.95 | −3.1(−3.51-−2.68) | 80.2(65.55-95.50) | −30.23 | −2.34(−2.71-−1.96) |
| Iceland | 1.96(1.7-2.25) | 39.83 | −1.13(−1.28-−0.98) | 23.61(19.59-27.95) | 39.28 | −0.94(−1.11-−0.77) |
| India | 42.38(31.68-53.52) | 55.43 | 0.21(0.14-0.27) | 638.92(491.99-804.47) | 84.50 | 0.28(0.22-0.34) |
| Indonesia | 10.26(8.34-12.43) | 40.55 | −0.05(−0.08-−0.01) | 148.85(124.74-177.75) | 75.27 | 0.24(0.22-0.26) |
| Iran  (Islamic Republic of) | 29.59(22.49-37.17) | 11.8 | −0.05(−0.07-−0.04) | 507.36(396.06-635.96) | 75.84 | 0.05(0.03-0.06) |
| Iraq | 32.19(24.58-40.52) | 132.98 | −0.07(−0.12-−0.02) | 524.3(410.3-659.23) | 182.84 | −0.01(−0.04-0.01) |
| Ireland | 2.61(2.28-2.97) | 18.8 | −1.8(−2-−1.6) | 30.74(25.52-36.33) | 34.81 | −1.22(−1.36-−1.07) |
| Israel | 3.59(3.2-4.04) | 92.77 | −0.9(−0.96-−0.84) | 36.34(30.44-43.02) | 87.18 | −0.9(−0.96-−0.84) |
| Italy | 6.45(5.56-7.37) | 34.77 | −0.59(−0.73-−0.45) | 73.24(63.24-82.72) | 40.84 | −0.6(−0.87-−0.32) |
| Jamaica | 48.6(36.62-62.09) | 1.52 | 0.16(0.15-0.18) | 874.77(681.52-1098.39) | 35.99 | 0.2(0.18-0.22) |
| Japan | 2.76(2.36-3.16) | 19.07 | −1.88(−2.01-−1.76) | 36.88(31.92-41.95) | 21.59 | −1.56(−1.69-−1.43) |
| Jordan | 1.71(1.46-1.99) | 184.36 | −1.6(−1.7-−1.51) | 21.94(17.95-26.52) | 231.45 | −0.89(−1-−0.79) |
| Kazakhstan | 7.1(6.26-7.99) | −35.56 | −2.8(−2.92-−2.68) | 103.42(87.79-120.94) | −18.3 | −1.75(−1.87-−1.63) |
| Kenya | 73.23(54.36-93.49) | 134.9 | 0.35(0.27-0.43) | 1082.8(838.02-1359.3) | 170.85 | 0.34(0.26-0.42) |
| Kiribati | 43.47(33.79-55.32) | 56.37 | 0.09(0.08-0.1) | 598.71(468.04-761.56) | 68.57 | 0.08(0.07-0.09) |
| Kuwait | 2.05(1.72-2.43) | 141.02 | −1.43(−1.55-−1.31) | 30.05(24.29-36.03) | 168.19 | −1.05(−1.15-−0.95) |
| Kyrgyzstan | 44.9(33.59-57.01) | 38.82 | 0.03(0-0.06) | 721.34(559.37-911.42) | 55.48 | 0.01(0-0.02) |
| Lao People's Democratic Republic | 40.92(31.28-51.69) | 58.77 | 0.07(−0.16-0.3) | 602.35(472.36-760.58) | 94.40 | 0.19(−0.04-0.41) |
| Latvia | 8.91(7.43-10.5) | −57.87 | −2.13(−2.25-−2) | 163.57(134.47-195.6) | −42.99 | −1.39(−1.6-−1.18) |
| Lebanon | 2.74(2.33-3.17) | 29.29 | −1.77(−1.88-−1.65) | 31.83(26.41-37.61) | 44.85 | −1.28(−1.4-−1.16) |
| Lesotho | 73.25(54.83-93.16) | 9.71 | −0.03(−0.03-−0.02) | 1018.7(786.95-1292.9) | 29.30 | 0.02(0.01-0.02) |
| Liberia | 58.75(44.49-75.01) | 173.97 | 0.23(0.18-0.27) | 843.66(651.65-1074.57) | 196.36 | 0.24(0.21-0.26) |
| Libya | 30.19(22.84-38.66) | 49.01 | 0.4(0.36-0.44) | 530.27(413.51-671.2) | 123.47 | 0.38(0.36-0.39) |
| Lithuania | 9.01(7.66-10.61) | −52.11 | −3.15(−3.36-−2.94) | 158.94(130.63-191.59) | −35.42 | −2.73(−2.95-−2.51) |
| Luxembourg | 3.95(3.41-4.57) | 44.16 | −0.42(−0.99-0.14) | 48.7(40.34-58.15) | 55.93 | −0.31(−0.94-0.33) |
| Madagascar | 79.83(60.15-102.57) | 138.59 | 0.23(0.21-0.25) | 1192.51(906.32-1509.08) | 155.40 | 0.22(0.19-0.24) |
| Malawi | 88.76(66.66-114.41) | 113.19 | 0.1(0.09-0.11) | 1246.94(952.92-1590.11) | 115.84 | 0.12(0.11-0.12) |
| Malaysia | 29.36(22.39-37.18) | 50.52 | −0.01(−0.04-0.02) | 545.99(427.84-687.27) | 96.00 | 0.1(0.07-0.12) |
| Maldives | 28.67(21.81-36.56) | 74.46 | −0.23(−0.25-−0.21) | 502.78(392.93-628.87) | 182.01 | −0.11(−0.13-−0.09) |
| Mali | 63.01(47.92-80.66) | 171.06 | 0.1(0.05-0.15) | 924.45(718.71-1173.12) | 166.86 | 0.13(0.08-0.19) |
| Malta | 2.34(2.07-2.64) | 38.03 | −1.92(−2.1-−1.74) | 20.77(17.17-25.1) | 48.5 | −1.77(−2.07-−1.47) |
| Marshall Islands | 41.54(31.97-52.81) | 4.18 | 0.06(0.05-0.07) | 607.8(474.5-775.47) | 33.89 | 0.08(0.07-0.09) |
| Mauritania | 52.95(39.92-67.76) | 99.85 | 0.04(0.03-0.05) | 833.36(649.28-1053.62) | 111.61 | 0.05(0.03-0.06) |
| Mauritius | 29.08(22.08-37.21) | −18.45 | −0.11(−0.12-−0.11) | 531.42(412.09-672.68) | 17.35 | 0(−0.01-0.01) |
| Mexico | 20.86(16.16-25.77) | 4.83 | −0.67(−0.72-−0.63) | 347.43(275.01-425.56) | 38.16 | −0.58(−0.61-−0.54) |
| Micronesia  (Federated States of) | 41.51(32.13-52.53) | −17.15 | 0.06(0.05-0.06) | 602.48(476.68-767.89) | 5.03 | 0.07(0.06-0.07) |
| Monaco | 3(2.48-3.6) | 26.38 | −0.46(−0.53-−0.38) | 38.55(31.65-47.11) | 25.37 | −0.38(−0.47-−0.29) |
| Mongolia | 47.48(35.91-59.98) | 24.08 | −0.13(−0.17-−0.09) | 748.64(581.26-941.97) | 70.65 | −0.01(−0.03-0.02) |
| Montenegro | 4.73(4.11-5.47) | 14.74 | −0.51(−0.58-−0.44) | 64.61(53.97-77.41) | 35.36 | −0.2(−0.34-−0.06) |
| Morocco | 32.84(25.11-41.91) | 21.93 | −0.05(−0.09-−0.02) | 533.66(413.34-674.57) | 60.06 | 0.03(0-0.06) |
| Mozambique | 88.25(65.59-112.83) | 140.74 | 0(−0.04-0.05) | 1254.35(962.78-1589.4) | 140.58 | 0.1(0.05-0.14) |
| Myanmar | 39.33(30.06-49.59) | 6.87 | −0.36(−0.42-−0.3) | 584.25(455.26-733.07) | 35.63 | −0.17(−0.25-−0.1) |
| Namibia | 66.8(49.7-87.25) | 58.2 | −0.06(−0.08-−0.05) | 1022.28(793.11-1308.37) | 82.19 | −0.03(−0.04-−0.02) |
| Nauru | 18.32(16.47-20.29) | −9.33 | −0.8(−0.98-−0.61) | 217.55(187.81-252.91) | −2.03 | −0.74(−0.89-−0.6) |
| Nepal | 36.37(27.4-45.69) | 63.01 | 0.14(0.07-0.21) | 534.1(410.56-669.54) | 87.07 | 0.2(0.14-0.27) |
| Netherlands | 2.62(2.25-3) | 68.82 | −0.14(−0.44-0.17) | 28.95(23.85-34.72) | 62.41 | −0.13(−0.33-0.07) |
| New Zealand | 4.69(4.1-5.34) | 33.94 | −1.26(−1.29-−1.22) | 56.09(49.55-62.94) | 46.69 | −0.58(−0.71-−0.46) |
| Nicaragua | 52.41(39.81-66.91) | 46.84 | 0.05(0.03-0.07) | 911.18(707.85-1139.33) | 98.37 | 0.12(0.1-0.14) |
| Niger | 55.87(42.12-71.15) | 217.5 | 0.22(0.19-0.24) | 800.22(621.24-1017.68) | 205.16 | 0.21(0.19-0.22) |
| Nigeria | 56.73(42.28-72) | 165.2 | 0.21(0.18-0.24) | 870.25(671.09-1094.65) | 163.96 | 0.3(0.27-0.33) |
| Niue | 11.96(10.5-13.42) | −33.46 | −1.12(−1.17-−1.07) | 171.31(146.04-198.52) | −18.75 | −0.46(−0.5-−0.42) |
| North Macedonia | 4.35(3.76-5.02) | −18.88 | −2.45(−2.59-−2.31) | 58.24(48.22-69.56) | 3.56 | −1.81(−1.95-−1.68) |
| Northern Mariana Islands | 36.52(27.78-46.98) | −33.28 | 0.22(0.2-0.24) | 662.48(526.02-840.51) | −6.94 | 0.14(0.12-0.16) |
| Norway | 2.16(1.79-2.52) | −13.4 | −3.41(−4.04-−2.77) | 21.7(17.98-25.41) | 3.45 | −2.71(−3.34-−2.07) |
| Oman | 2.59(2.21-3.01) | 136.04 | −0.8(−0.87-−0.74) | 32.01(26.16-38.34) | 180.35 | −0.18(−0.22-−0.14) |
| Pakistan | 51.81(39.03-65.25) | 117.07 | 0.14(−0.03-0.31) | 770.7(593.54-962.81) | 134.12 | 0.19(0.02-0.36) |
| Palau | 9.47(8.41-10.62) | 33.82 | −1.01(−1.07-−0.95) | 139.63(119.38-160.87) | 55.55 | −0.56(−0.63-−0.5) |
| Palestine | 29.44(22.49-37.38) | 134.8 | −0.01(−0.02-−0.01) | 525.1(409.5-663.68) | 183.58 | 0.05(0.04-0.05) |
| Panama | 45.54(34.22-57.44) | 40.4 | −0.16(−0.17-−0.14) | 842.14(655.8-1049.65) | 76.51 | −0.01(−0.02-0.01) |
| Papua New Guinea | 41.33(32.29-52.61) | 141.5 | 0.16(0.13-0.18) | 588.27(468.22-751.72) | 156.34 | 0.18(0.16-0.2) |
| Paraguay | 49.2(36.82-62.99) | 49.51 | −0.05(−0.06-−0.04) | 877.75(679.3-1099.81) | 90.53 | 0.01(0-0.02) |
| Peru | 46.78(35.24-59.22) | 29.79 | −0.02(−0.03-−0.01) | 814.32(631.82-1013.24) | 71.59 | 0.1(0.08-0.12) |
| Philippines | 25.51(19.49-32.54) | 75.88 | 0.47(0.36-0.57) | 398.21(308.43-502.12) | 108.18 | 0.52(0.41-0.62) |
| Poland | 5.82(4.92-6.68) | −43.24 | −2.73(−2.96-−2.5) | 81.44(68.51-94.04) | −20.53 | −1.63(−1.79-−1.47) |
| Portugal | 3.05(2.71-3.45) | −11.71 | −2.4(−2.59-−2.2) | 34.17(28.93-39.96) | −5.71 | −2.13(−2.36-−1.91) |
| Puerto Rico | 2.59(2.15-3.04) | 30.39 | −1.1(−1.22-−0.99) | 36.9(30.58-43.97) | 45.72 | −0.66(−0.75-−0.58) |
| Qatar | 2.94(2.47-3.5) | 376.94 | −1.28(−1.44-−1.11) | 37.79(30.78-45.4) | 427.7 | −1.13(−1.22-−1.04) |
| Republic of Korea | 1.53(1.3-1.79) | 72.19 | −1.38(−1.53-−1.23) | 25.08(20.75-29.76) | 97.85 | −0.85(−0.99-−0.72) |
| Republic of Moldova | 6.06(5.24-7.05) | −54.67 | −3.13(−3.21-−3.06) | 98.2(82.01-116.59) | −43.34 | −2.51(−2.74-−2.28) |
| Romania | 5.81(5.03-6.74) | −47.06 | −2.23(−2.32-−2.15) | 86.64(71.6-103.52) | −29.82 | −1.32(−1.53-−1.11) |
| Russian Federation | 8.54(7.46-9.71) | −38.03 | −2.37(−2.5-−2.23) | 145.11(126.21-166.26) | −26.26 | −1.82(−2.04-−1.6) |
| Rwanda | 79.41(59.65-102.68) | 76.44 | −0.01(−0.02-0) | 1157.46(890.3-1470.83) | 99.42 | 0.04(0.03-0.05) |
| Saint Kitts and Nevis | 3.08(2.66-3.51) | −4.31 | −2.38(−2.53-−2.24) | 40.21(33.64-46.97) | 8.99 | −1.93(−2.07-−1.8) |
| Saint Lucia | 50.67(37.94-64.7) | −11.37 | −0.03(−0.05-−0.01) | 894.68(692.4-1133.42) | 41.14 | 0.01(0-0.02) |
| Saint Vincent and the Grenadines | 50.95(38.2-64.67) | −26.76 | −0.09(−0.11-−0.07) | 856.11(669.22-1071.49) | 7.94 | 0(−0.02-0.01) |
| Samoa | 40.15(30.66-51.43) | 20.14 | 0.04(0.02-0.06) | 644.37(508.77-818.04) | 34.77 | 0.1(0.09-0.11) |
| San Marino | 4.68(4.04-5.34) | 47.42 | −0.99(−1.06-−0.91) | 45.44(37.3-54.35) | 46.18 | −0.78(−0.81-−0.75) |
| Sao Tome and Principe | 54.82(40.82-69.56) | 75.97 | 0.3(0.27-0.33) | 895.37(690.82-1122.71) | 109.94 | 0.32(0.3-0.35) |
| Saudi Arabia | 2.8(2.44-3.22) | 119.03 | −0.78(−0.97-−0.59) | 35.91(30.12-42.46) | 137.05 | −0.58(−0.72-−0.44) |
| Senegal | 55.34(41.35-71.66) | 102.7 | 0.03(−0.02-0.07) | 847.35(653.46-1082.19) | 123.96 | 0.03(−0.01-0.08) |
| Serbia | 4.14(3.56-4.78) | −29.97 | −1.75(−1.86-−1.64) | 55.72(45.97-66.41) | −4.37 | −1.2(−1.33-−1.06) |
| Seychelles | 29.1(21.85-37.05) | 4.71 | −0.17(−0.18-−0.16) | 522.5(407.39-667.27) | 43.45 | −0.15(−0.16-−0.14) |
| Sierra Leone | 58.7(44.03-74.83) | 152.37 | 0.19(0.14-0.24) | 842.54(646.47-1071.13) | 157.59 | 0.18(0.14-0.21) |
| Singapore | 1.61(1.33-1.96) | 24.77 | −3.36(−3.67-−3.06) | 28.3(23.28-33.91) | 50.29 | −2.82(−3.08-−2.56) |
| Slovakia | 7.12(5.95-8.48) | 44.09 | 0(−0.34-0.33) | 111.4(91.01-134.24) | 69.32 | 0.29(−0.16-0.74) |
| Slovenia | 5.71(4.99-6.57) | 12.3 | −1.97(−2.21-−1.74) | 63.05(51.33-77.04) | 25.96 | −2.16(−2.58-−1.74) |
| Solomon Islands | 43.51(33.67-54.87) | 85.83 | 0.08(0.07-0.09) | 593.17(463.92-751.36) | 103.91 | 0.08(0.08-0.09) |
| Somalia | 89.01(66.29-113.84) | 205.74 | 0.22(0.21-0.23) | 1216.61(930.29-1547.05) | 209.17 | 0.18(0.17-0.19) |
| South Africa | 73.88(54.94-93.23) | 30.83 | 0(−0.01-0.02) | 1120.29(865.81-1415.5) | 58.69 | 0.03(0.01-0.05) |
| South Sudan | 69.66(51.88-88.51) | 66.15 | 0.14(0.13-0.15) | 1147.98(895.27-1434.66) | 64.6 | 0.15(0.14-0.16) |
| Spain | 4.45(3.95-4.97) | 4.02 | −2.23(−2.33-−2.13) | 40.68(33.9-48.11) | −1.21 | −2.17(−2.29-−2.06) |
| Sri Lanka | 4.8(4.09-5.6) | 34.74 | −1.44(−1.56-−1.31) | 77.4(65.05-91.9) | 66.9 | −0.95(−1.07-−0.83) |
| Sudan | 36.11(27.5-45.59) | 113.22 | 0.05(0.02-0.07) | 549.19(426.14-693.39) | 134 | 0.15(0.13-0.17) |
| Suriname | 49.15(36.57-62.21) | 21.68 | −0.07(−0.09-−0.05) | 818.31(638.26-1017.59) | 53.53 | 0.02(0.01-0.03) |
| Sweden | 2.21(1.86-2.56) | −13.33 | −2.23(−2.56-−1.9) | 22.12(18.55-25.65) | −5.41 | −2.04(−2.46-−1.62) |
| Switzerland | 3(2.52-3.5) | −22.49 | −2.69(−2.84-−2.55) | 38.85(31.62-46.81) | −2.94 | −1.81(−2.04-−1.57) |
| Syrian Arab Republic | 30.93(23.72-39.53) | −1.77 | 0.01(−0.02-0.05) | 521.39(407.43-657.44) | 37.43 | 0.07(0.04-0.09) |
| Taiwan  (Province of China) | 9.11(8.04-10.12) | 57.48 | −1.35(−1.57-−1.14) | 148.36(131.93-168.37) | 101.31 | −0.67(−0.84-−0.5) |
| Tajikistan | 46.37(35-58.71) | 70.49 | 0.07(0.01-0.13) | 741.95(577.37-935.32) | 99.42 | 0.04(0.01-0.06) |
| Thailand | 28.84(22.02-36.63) | −21.42 | −0.18(−0.2-−0.16) | 481.42(376.67-607.31) | 22.37 | −0.08(−0.09-−0.06) |
| Timor-Leste | 34.11(26.08-43.83) | 70.62 | −0.01(−0.03-0.01) | 522.78(408.88-664.28) | 80.94 | 0.13(0.12-0.13) |
| Togo | 57.64(43.49-73.41) | 116.57 | 0.21(0.19-0.23) | 832.59(645.55-1056.34) | 146.92 | 0.19(0.17-0.21) |
| Tokelau | 13.67(12.22-15.27) | −34.97 | −1.6(−1.65-−1.54) | 174.82(150.39-201.97) | −17.86 | −0.69(−0.73-−0.66) |
| Tonga | 52.78(40.83-67.04) | −3.05 | −0.18(−0.6-0.24) | 929.25(737.21-1170.76) | 8.67 | −0.14(−0.54-0.27) |
| Trinidad and Tobago | 48.9(36.62-61.95) | −18.53 | −0.16(−0.18-−0.13) | 866.42(673.13-1079.27) | 19.64 | 0.01(0-0.02) |
| Tunisia | 2.57(2.23-2.92) | 54.09 | −1.24(−1.31-−1.16) | 28.75(23.86-33.92) | 68.41 | −0.75(−0.82-−0.69) |
| Turkey | 2.21(1.91-2.53) | 46.07 | −1.46(−1.72-−1.21) | 27.99(23.1-33.26) | 75.42 | −0.82(−1.03-−0.61) |
| Turkmenistan | 43.46(32.83-55.34) | 14.53 | −0.11(−0.15-−0.06) | 718.11(555.73-906.94) | 48.23 | 0.01(0-0.02) |
| Tuvalu | 17.32(15.49-19.17) | −3.55 | −1.38(−1.44-−1.33) | 180.15(153.76-210.24) | 22.53 | −0.73(−0.82-−0.65) |
| Uganda | 93.95(68.41-128.32) | 176.33 | 0.12(−0.05-0.29) | 1348.18(1015.01-1827.17) | 184.12 | 0.18(0.01-0.35) |
| Ukraine | 6.38(5.54-7.31) | −32.28 | −1.13(−1.21-−1.06) | 104.66(90.11-121.42) | −26.11 | −0.87(−1.08-−0.66) |
| United Arab Emirates | 27.57(20.94-34.7) | 278.52 | 0.06(0.02-0.09) | 511.64(397.8-640.2) | 494.57 | −0.06(−0.09-−0.03) |
| United Kingdom | 2.49(2.14-2.84) | −34.86 | −3(−3.19-−2.81) | 26.79(22.8-30.94) | −26.26 | −2.37(−2.55-−2.2) |
| United Republic of Tanzania | 81.02(60.37-103.32) | 126.08 | 0.15(0.14-0.16) | 1200.88(925.33-1515.12) | 142.15 | 0.22(0.21-0.23) |
| United States of America | 9.45(8.59-10.38) | 69.18 | 0.22(−0.07-0.51) | 118.33(108.81-127.92) | 67.1 | 0.09(−0.12-0.29) |
| United States Virgin Islands | 3.14(2.64-3.67) | 51.78 | −0.34(−0.44-−0.24) | 45.04(37.72-53.07) | 63.81 | −0.18(−0.32-−0.04) |
| Uruguay | 4.53(3.98-5.2) | 19.64 | −0.67(−0.84-−0.5) | 56.95(47.94-66.68) | 35.11 | −0.2(−0.37-−0.02) |
| Uzbekistan | 45.89(34.73-58.31) | 51.53 | 0.19(0.18-0.21) | 746.93(575.56-937.72) | 91.02 | 0.31(0.28-0.34) |
| Vanuatu | 45.29(34.96-57.29) | 82.62 | 0.03(0.02-0.04) | 666.76(524.47-850.63) | 103.58 | 0.04(0.04-0.05) |
| Venezuela  (Bolivarian Republic of) | 2.56(2.19-2.98) | 51.63 | −1.52(−1.76-−1.28) | 34.85(29.33-41.36) | 64.79 | −1.24(−1.47-−1.02) |
| Viet Nam | 7.62(6.54-8.7) | 109.4 | −0.11(−0.41-0.19) | 119.78(101.09-138.21) | 141.61 | 0.68(0.38-0.97) |
| Yemen | 42.93(32.89-53.99) | 153 | 0.25(−0.05-0.55) | 639.56(495.8-803.27) | 188.51 | 0.28(−0.02-0.59) |
| Zambia | 81.63(60.76-103.95) | 123.57 | −0.12(−0.15-−0.09) | 1168.6(891.08-1479.12) | 141.96 | −0.08(−0.11-−0.04) |
| Zimbabwe | 71.64(53.44-91.3) | 44.21 | 0.19(0.16-0.22) | 1002.73(765.13-1271.83) | 59.16 | 0.12(0.1-0.15) |

RHD: rheumatic heart disease; EAPC: estimated annual percentage change; ASR, age-standardized rate; CI, confidence interval; UI: uncertainty interval.

**Supplementary table 3**. The age-standardized rate of death and DALYs caused by RHD at national level and both sexes in 2019, and percentage changes and EAPCs from 1990 to 2019.

|  | **Death** | | | **DALYs** | | |
| --- | --- | --- | --- | --- | --- | --- |
| **Characteristics** | ASR/100,000  (95% UI) | Percentage change (%) | EAPC  (95%CI) | ASR/100,000  (95% UI) | Percentage change (%) | EAPC  (95%CI) |
| Afghanistan | 6.15(3.61-9.65) | 20.46 | −2.26(−2.55-−1.96) | 210.71(141.02-302.17) | 44.74 | −2.4(−2.69-−2.11) |
| Albania | 1.06(0.76-1.42) | −62.61 | −5.7(−6.16-−5.24) | 64.77(46.74-85.31) | −57.3 | −3.34(−3.72-−2.97) |
| Algeria | 1.39(1.12-1.69) | −22.72 | −3.51(−3.58-−3.44) | 54.4(43.02-67.07) | −25.31 | −3.26(−3.34-−3.17) |
| American Samoa | 5.69(4.56-7.13) | 9.83 | −1.57(−1.74-−1.39) | 223.57(179.24-276.62) | −1.7 | −1.21(−1.35-−1.07) |
| Andorra | 0.97(0.72-1.27) | 96.37 | −1.98(−2.31-−1.65) | 18.56(14.06-24.15) | 46.34 | −1.98(−2.28-−1.68) |
| Angola | 3.24(2.23-4.26) | 14.67 | −2.9(−3.11-−2.69) | 142.96(108.35-186.26) | 52.26 | −2.4(−2.55-−2.26) |
| Antigua and Barbuda | 1.01(0.84-1.21) | −19.46 | −3.01(−3.17-−2.85) | 73.7(55.31-97.78) | 2.78 | −1.85(−1.96-−1.74) |
| Argentina | 2.87(2.34-3.46) | −19.71 | −3.17(−3.29-−3.06) | 78.71(63.06-98.09) | −23.98 | −2.73(−2.85-−2.6) |
| Armenia | 2.81(2.25-3.42) | −45.8 | −3.46(−3.81-−3.11) | 111(87.75-137.12) | −53.39 | −3.44(−3.75-−3.13) |
| Australia | 1.03(0.87-1.21) | 9.02 | −2.43(−2.84-−2.02) | 22(19.03-25.33) | −14.95 | −2.76(−3.13-−2.38) |
| Austria | 1.45(1.2-1.69) | −10.27 | −2.35(−2.65-−2.04) | 23.36(20.48-26.47) | −37.39 | −3.37(−3.65-−3.08) |
| Azerbaijan | 2.06(1.58-2.69) | −23.36 | −2.52(−2.72-−2.33) | 95.52(72.84-121.25) | −13.65 | −2.51(−2.64-−2.38) |
| Bahamas | 0.93(0.69-1.23) | 2.87 | −2.55(−2.7-−2.41) | 75.31(55.46-100.9) | 14.81 | −1.4(−1.51-−1.3) |
| Bahrain | 1.12(0.87-1.41) | 82.76 | −2.99(−3.21-−2.78) | 25.72(20.6-31.93) | 57.66 | −3.62(−3.89-−3.34) |
| Bangladesh | 2.74(1.93-3.57) | 13.96 | −2.21(−2.77-−1.64) | 105.36(82.09-130.38) | −0.79 | −2.11(−2.49-−1.73) |
| Barbados | 1.03(0.79-1.3) | −23.94 | −2.55(−2.68-−2.42) | 75.24(56.13-100.42) | −16.29 | −1.53(−1.63-−1.43) |
| Belarus | 1.7(1.19-2.4) | −69.96 | −5.54(−5.93-−5.14) | 58.35(42.12-80.55) | −72.06 | −5.75(−6.12-−5.37) |
| Belgium | 1.66(1.31-2.01) | 174.55 | 2.24(1.70-2.77) | 24.35(20.55-28.35) | 87.95 | 1.14(0.75-1.54) |
| Belize | 1.07(0.84-1.34) | 56.31 | −2.53(−2.86-−2.19) | 80.27(60.91-102.34) | 89.7 | −1.5(−1.71-−1.29) |
| Benin | 2.81(2.16-3.67) | −0.57 | −2.89(−3.08-−2.7) | 115.65(88.35-148.89) | 39.19 | −2.19(−2.36-−2.03) |
| Bermuda | 0.45(0.36-0.58) | −50.96 | −4.71(−4.94-−4.47) | 15.86(12.66-20.24) | −60.44 | −4.62(−4.88-−4.36) |
| Bhutan | 12.5(6.04-25.04) | −1.57 | −2.55(−2.62-−2.47) | 338.5(182.35-639.07) | −22.69 | −2.95(−3.04-−2.85) |
| Bolivia  (Plurinational State of) | 2.75(1.97-3.65) | −9.73 | −3.51(−3.64-−3.37) | 102.93(77.08-132.02) | −3.75 | −3.02(−3.16-−2.89) |
| Bosnia and Herzegovina | 0.6(0.48-0.76) | −63.01 | −5.82(−6.15-−5.48) | 15.56(12.43-19.36) | −67.25 | −5.75(−6.11-−5.39) |
| Botswana | 2.25(1.15-3.26) | −14.33 | −3.58(−3.82-−3.35) | 118.4(80.19-159.67) | 12.68 | −2.62(−2.78-−2.47) |
| Brazil | 1.16(1.07-1.24) | −12.08 | −3.07(−3.17-−2.96) | 79.34(61.58-102.55) | −6.82 | −2.05(−2.09-−2.01) |
| Brunei Darussalam | 2.65(2.28-3.04) | 39.43 | −1.63(−1.9-−1.35) | 58.27(50.23-67.04) | 18.94 | −2.06(−2.28-−1.84) |
| Bulgaria | 1.71(1.24-2.32) | −72.72 | −5.36(−5.88-−4.84) | 56.35(40.98-75.04) | −75.4 | −5.39(−5.92-−4.86) |
| Burkina Faso | 2.9(2.25-3.6) | 53.82 | −0.94(−1.01-−0.88) | 115.7(89.51-145.43) | 81.37 | −0.8(−0.88-−0.72) |
| Burundi | 3.05(2.09-4.22) | −13.01 | −2.8(−2.96-−2.63) | 144.71(106.65-191.12) | 16.1 | −2.27(−2.41-−2.13) |
| Cabo Verde | 1.3(0.95-1.57) | −66.05 | −5.72(−6.68-−4.76) | 74.5(54.92-99.82) | −38.06 | −3.68(−4.38-−2.97) |
| Cambodia | 2.71(1.76-3.73) | −27.27 | −3.79(−3.91-−3.66) | 119.69(84.58-163.54) | −30.64 | −3.68(−3.81-−3.55) |
| Cameroon | 2.32(1.62-3.33) | 4.62 | −3.31(−3.39-−3.23) | 98.58(71.73-133.16) | 65.41 | −2.26(−2.32-−2.2) |
| Canada | 1.05(0.85-1.25) | 17.07 | −2.59(−2.81-−2.37) | 23.49(19.78-27.74) | 0.92 | −2.61(−2.84-−2.38) |
| Central African Republic | 7.47(4.14-12.32) | 30.11 | −1.24(−1.32-−1.16) | 270.11(173.98-406.56) | 44.56 | −1.15(−1.22-−1.08) |
| Chad | 3.93(2.94-5.6) | 11.8 | −2.01(−2.07-−1.95) | 144.61(111.68-191.55) | 51.49 | −1.59(−1.65-−1.54) |
| Chile | 0.81(0.67-0.97) | −57.88 | −6.28(−6.58-−5.98) | 23.4(19.72-28.02) | −64.4 | −6.3(−6.55-−6.06) |
| China | 4.04(3.32-4.69) | −47.4 | −5.05(−5.17-−4.93) | 93.73(78.43-108.78) | −57.56 | −5.22(−5.31-−5.12) |
| Colombia | 0.38(0.28-0.52) | −54.1 | −6.19(−6.44-−5.93) | 12.09(9.18-15.73) | −63.98 | −6.18(−6.44-−5.93) |
| Comoros | 2.12(1.49-2.94) | −7.41 | −2.66(−2.88-−2.43) | 112.79(83.14-148.51) | 7.59 | −1.93(−2.16-−1.71) |
| Congo | 2.82(2.02-3.78) | −10.41 | −3.35(−3.55-−3.15) | 129.05(95.57-170.24) | 26.45 | −2.67(−2.85-−2.5) |
| Cook Islands | 2.11(1.62-2.67) | −34.53 | −2.57(−2.74-−2.39) | 77.54(56.13-101.7) | −48.28 | −2.55(−2.73-−2.38) |
| Costa Rica | 0.97(0.71-1.3) | 2.7 | −3.44(−3.65-−3.22) | 75.56(54.96-101.84) | 19.53 | −1.85(−2-−1.7) |
| Croatia | 1.11(0.85-1.43) | −59.67 | −3.79(−4.72-−2.85) | 23.42(18.45-29.73) | −68.23 | −4.46(−5.32-−3.6) |
| Cuba | 1.08(0.82-1.4) | −31.43 | −2.97(−3.24-−2.7) | 79.36(59.51-104.33) | −33.98 | −2.11(−2.25-−1.96) |
| Cyprus | 2.77(2.33-3.38) | −7.62 | −3.84(−4.15-−3.52) | 41.41(35.39-48.88) | −20.79 | −4.24(−4.52-−3.96) |
| Czechia | 1.35(1.07-1.68) | −59.41 | −4.41(−5.16-−3.66) | 28.27(22.68-34.44) | −70.4 | −5.35(−6.09-−4.61) |
| Cote d'Ivoire | 2.31(1.77-3.01) | 4.54 | −2.74(−2.87-−2.61) | 99.51(74.24-128.37) | 34.89 | −1.92(−2.03-−1.81) |
| Democratic People's Republic of Korea | 7.9(4.81-11.54) | 32.92 | −1.53(−1.82-−1.25) | 206.87(133.72-297.64) | 14.14 | −1.46(−1.7-−1.23) |
| Democratic Republic of the Congo | 4.16(2.39-7.39) | 29.29 | −1.48(−1.72-−1.23) | 165.39(113.04-252.77) | 51.9 | −1.34(−1.5-−1.18) |
| Denmark | 0.69(0.54-0.83) | −66.17 | −3.73(−5.01-−2.43) | 11.58(9.56-13.59) | −71.75 | −4.51(−5.63-−3.37) |
| Djibouti | 1.81(1.18-2.54) | 48.41 | −2.63(−2.76-−2.5) | 104.93(72.56-145.1) | 82.84 | −1.65(−1.75-−1.56) |
| Dominica | 1.84(1.43-2.38) | −40.27 | −2.62(−2.74-−2.51) | 103.38(79.08-131.07) | −33.7 | −1.89(−1.97-−1.81) |
| Dominican Republic | 1.46(0.93-2.02) | 6.83 | −1.55(−1.86-−1.23) | 89.22(65.41-118.92) | 2.51 | −1.33(−1.55-−1.11) |
| Ecuador | 1.31(1.04-1.65) | −15.7 | −3.62(−3.86-−3.38) | 69.55(51.84-91.22) | 7.34 | −2.32(−2.51-−2.14) |
| Egypt | 1.93(1.19-2.8) | −47.88 | −3.29(−3.53-−3.04) | 84.67(58.98-114.97) | −49.93 | −3.49(−3.8-−3.17) |
| El Salvador | 0.59(0.43-0.78) | −30.38 | −3.83(−4.13-−3.53) | 56.52(39.01-78.79) | −8.58 | −1.65(−1.81-−1.5) |
| Equatorial Guinea | 1.72(1.03-2.61) | −53.51 | −6.05(−6.48-−5.62) | 95.2(66.22-133.43) | 18.93 | −4.45(−4.84-−4.06) |
| Eritrea | 3.53(2.6-4.71) | 17.49 | −2.04(−2.11-−1.97) | 160.06(123.37-208.95) | 41.56 | −1.63(−1.73-−1.53) |
| Estonia | 0.96(0.67-1.33) | −76.9 | −6.95(−7.43-−6.47) | 33.29(25.31-44.27) | −77.66 | −6.79(−7.26-−6.32) |
| Eswatini | 2.91(2.06-3.99) | −11.35 | −2.03(−2.45-−1.61) | 140.41(103.49-187.11) | 3.66 | −1.45(−1.77-−1.14) |
| Ethiopia | 2.34(1.61-2.97) | −37.9 | −3.91(−4.06-−3.76) | 112.09(83.99-145.89) | −9.61 | −3.23(−3.33-−3.13) |
| Fiji | 8.76(6.9-11.18) | −11.24 | −1.86(−1.95-−1.78) | 334.71(263.46-425.65) | −17.6 | −1.67(−1.72-−1.62) |
| Finland | 0.39(0.32-0.48) | −61.22 | −5.39(−5.59-−5.19) | 7.76(6.58-9.3) | −66.95 | −5.5(−5.74-−5.26) |
| France | 1.44(1.15-1.74) | 18.65 | −1.91(−2.29-−1.54) | 24.27(20.53-28.12) | −10.67 | −2.28(−2.61-−1.96) |
| Gabon | 1.87(1.35-2.52) | −34.42 | −3.45(−3.62-−3.28) | 100.76(73.2-136.62) | 8.73 | −2.3(−2.37-−2.22) |
| Gambia | 2.58(1.83-3.38) | 29 | −2.31(−2.55-−2.08) | 106.11(80.03-136.06) | 52.76 | −1.62(−1.8-−1.44) |
| Georgia | 6.03(4.84-7.33) | 55.34 | 2.64(2.12-3.16) | 182.3(150.69-215.41) | −18.22 | 0.49(0.19-0.79) |
| Germany | 1.98(1.66-2.35) | 4.34 | −1.78(−2.09-−1.47) | 30.05(26.11-34.82) | −29.34 | −2.84(−3.28-−2.41) |
| Ghana | 1.8(1.44-2.25) | 12.96 | −2.35(−2.42-−2.28) | 84.49(64.31-108.98) | 47.97 | −1.56(−1.61-−1.51) |
| Greece | 0.57(0.47-0.69) | −24.45 | −4.07(−4.88-−3.26) | 11.06(9.26-13.07) | −38.15 | −3.72(−4.42-−3.01) |
| Greenland | 1.54(1.27-1.85) | −39.17 | −5.36(−5.8-−4.92) | 35.42(29.44-42.17) | −42.66 | −4.91(−5.27-−4.55) |
| Grenada | 2.36(1.92-2.88) | −43.73 | −3.16(−3.38-−2.93) | 120.82(98.06-149.19) | −32.73 | −2.6(−2.81-−2.39) |
| Guam | 2.37(1.96-2.88) | −2.62 | −2.94(−3.29-−2.59) | 125.89(104.04-151.28) | −6.25 | −1.6(−1.77-−1.43) |
| Guatemala | 0.52(0.39-0.67) | −1.63 | −3.46(−3.9-−3.02) | 53.61(37.11-76.03) | 76.1 | −1.26(−1.54-−0.98) |
| Guinea | 3.61(2.7-4.83) | −20.79 | −2.32(−2.5-−2.14) | 131.37(100.23-169.56) | 1.08 | −1.86(−2.04-−1.69) |
| Guinea−Bissau | 4.37(3.15-5.81) | −31.69 | −3(−3.17-−2.84) | 160.43(119.02-203.67) | −14.39 | −2.55(−2.7-−2.4) |
| Guyana | 2.13(1.55-2.81) | −38.24 | −2.48(−2.58-−2.39) | 122.93(93.49-157.17) | −34.51 | −1.83(−1.94-−1.73) |
| Haiti | 7.89(4.53-12.79) | −5.63 | −2.5(−2.65-−2.35) | 341.78(212.66-524.06) | −5.44 | −2.45(−2.62-−2.28) |
| Honduras | 0.82(0.44-1.13) | 98.7 | −0.97(−1.14-−0.8) | 58.64(40.65-80.5) | 115.55 | −0.53(−0.57-−0.48) |
| Hungary | 1.34(1.06-1.64) | −70.78 | −5.69(−6.41-−4.97) | 29.74(24.12-35.98) | −77.35 | −6.46(−7.16-−5.76) |
| Iceland | 0.49(0.39-0.58) | −1.29 | −2.78(−2.9-−2.67) | 9.9(8.28-11.65) | −14.31 | −2.85(−2.97-−2.72) |
| India | 11.51(8.63-14.03) | 24.07 | −2.38(−2.52-−2.25) | 350.7(271.91-420.8) | 7.82 | −2.28(−2.4-−2.16) |
| Indonesia | 0.67(0.57-0.76) | −49.11 | −4.34(−4.68-−3.99) | 26.05(22.24-30.53) | −47.77 | −4.04(−4.29-−3.78) |
| Iran  (Islamic Republic of) | 1.48(1.26-1.67) | −9.34 | −3.24(−3.67-−2.8) | 61.29(51.34-74.5) | −21.39 | −2.84(−3.15-−2.53) |
| Iraq | 1.53(1.19-1.9) | −27.53 | −5.02(−5.47-−4.56) | 64.61(50.95-80.63) | −2.83 | −4.32(−4.68-−3.96) |
| Ireland | 0.81(0.65-0.99) | −32.39 | −3.36(−3.65-−3.06) | 16.24(13.44-19.46) | −40.63 | −3.68(−3.95-−3.4) |
| Israel | 1.63(1.37-1.89) | 55.5 | −1.67(−1.87-−1.48) | 33.3(28.53-38.89) | 19.91 | −2.25(−2.4-−2.1) |
| Italy | 1.59(1.38-1.74) | 2.22 | −2.29(−2.43-−2.15) | 31.27(28.36-34.01) | −31.58 | −3.13(−3.32-−2.94) |
| Jamaica | 1.08(0.78-1.42) | −47.07 | −3.58(−4.27-−2.88) | 87.37(64.91-114.04) | −33.98 | −2.27(−2.73-−1.81) |
| Japan | 0.88(0.68-1) | 58.35 | −2.92(−2.98-−2.85) | 14.13(12.32-15.58) | −9.7 | −3.53(−3.63-−3.42) |
| Jordan | 0.45(0.35-0.56) | 16.22 | −4.79(−4.98-−4.6) | 12.68(10.23-15.39) | 6.98 | −4.96(−5.18-−4.73) |
| Kazakhstan | 1.99(1.51-2.53) | −65.99 | −5.83(−6.41-−5.26) | 68.09(52.27-86.59) | −69.72 | −6.31(−6.94-−5.68) |
| Kenya | 1.84(0.98-2.82) | 53.32 | −1.13(−1.27-−0.98) | 96.13(66.19-132.46) | 100.58 | −0.57(−0.68-−0.46) |
| Kiribati | 18.8(13.89-24.24) | 6.9 | −1.59(−1.68-−1.51) | 733.8(543.56-967.27) | 6.15 | −1.65(−1.73-−1.58) |
| Kuwait | 0.43(0.33-0.55) | −26.64 | −4.43(−4.94-−3.92) | 12.01(9.64-14.99) | −37.51 | −4.62(−5.16-−4.07) |
| Kyrgyzstan | 3.05(2.39-3.78) | −45.08 | −4.37(−4.72-−4.02) | 148.61(119.99-181.13) | −40.09 | −4.14(−4.43-−3.84) |
| Lao People's Democratic Republic | 3.58(2.51-4.72) | −5.05 | −2.69(−2.89-−2.48) | 168.46(122.05-219.98) | 1.5 | −2.26(−2.51-−2.02) |
| Latvia | 1.13(0.84-1.53) | −83.32 | −7.16(−7.57-−6.75) | 41.11(31.73-54.13) | −84.03 | −7(−7.41-−6.58) |
| Lebanon | 0.84(0.39-1.28) | −27.89 | −3.99(−4.16-−3.82) | 24.6(12.67-36.19) | −41.84 | −4.15(−4.37-−3.92) |
| Lesotho | 4.38(3.05-6.09) | −10.38 | −0.57(−0.85-−0.3) | 184.56(136.57-246.51) | −0.31 | −0.33(−0.58-−0.08) |
| Liberia | 2.47(1.56-3.94) | −26.78 | −3.29(−3.51-−3.07) | 102.34(72.81-140.99) | 13.79 | −2.54(−2.77-−2.31) |
| Libya | 1.05(0.74-1.43) | −33.53 | −3.06(−3.38-−2.74) | 61.36(45.19-81.45) | −31.82 | −2.69(−2.95-−2.44) |
| Lithuania | 1.36(1.02-1.76) | −78.7 | −6.55(−6.85-−6.25) | 46.18(35.84-58.07) | −80.3 | −6.56(−6.86-−6.25) |
| Luxembourg | 1.05(0.84-1.29) | 0.73 | −2.46(−2.52-−2.4) | 20.62(17.03-24.46) | −13.24 | −2.62(−2.71-−2.54) |
| Madagascar | 4.22(2.93-5.65) | 11.07 | −1.64(−1.7-−1.59) | 186.58(140.26-243) | 25.96 | −1.58(−1.7-−1.46) |
| Malawi | 2.12(1.57-2.68) | −13.7 | −2.47(−2.6-−2.34) | 116.08(87.71-151.83) | 18.51 | −1.83(−1.92-−1.74) |
| Malaysia | 0.85(0.62-1.08) | −52.11 | −5.09(−5.41-−4.77) | 52.65(39.6-67.44) | −32.43 | −3.56(−3.91-−3.2) |
| Maldives | 1.21(0.98-1.45) | −46.75 | −5.71(−6.05-−5.36) | 56.37(43.54-72.13) | −36.76 | −5.06(−5.4-−4.72) |
| Mali | 3.56(2.53-5.12) | −14.37 | −3.26(−3.52-−3.01) | 140.31(103.64-185.07) | 16.76 | −2.65(−2.89-−2.41) |
| Malta | 1.02(0.83-1.24) | 6.82 | −2.71(−2.79-−2.63) | 19.9(16.46-23.97) | −10.65 | −2.71(−2.77-−2.64) |
| Marshall Islands | 14.57(9.31-21.11) | 24.05 | −1.4(−1.56-−1.25) | 572.74(382.76-822.14) | 22.14 | −1.08(−1.27-−0.9) |
| Mauritania | 1.65(1.2-2.19) | −44.75 | −4.05(−4.29-−3.81) | 79.87(59.48-105.54) | −7.99 | −2.78(−2.99-−2.56) |
| Mauritius | 0.82(0.63-1.06) | −59.89 | −4.28(−5-−3.55) | 53.74(40.54-69.82) | −53.53 | −2.94(−3.51-−2.37) |
| Mexico | 0.88(0.75-1.03) | −43.72 | −5.29(−5.51-−5.07) | 40.61(33.08-51.1) | −39.9 | −4.38(−4.62-−4.15) |
| Micronesia  (Federated States of) | 13.69(8.49-20.53) | −24.24 | −1.96(−2.07-−1.86) | 513.68(302.11-765.91) | −26.22 | −1.82(−1.9-−1.74) |
| Monaco | 0.55(0.42-0.66) | −6.81 | −1.35(−1.41-−1.29) | 11.71(9.23-14.2) | −14.57 | −1.48(−1.54-−1.42) |
| Mongolia | 5.42(4.25-6.78) | −13.85 | −3.93(−4.34-−3.5) | 175.53(138.39-221.84) | 1.23 | −3.59(−3.96-−3.22) |
| Montenegro | 1.26(0.97-1.56) | −0.52 | −1.66(−1.99-−1.34) | 34.99(27.79-43.07) | −14.46 | −2.05(−2.39-−1.7) |
| Morocco | 2.19(1.64-2.87) | −17.95 | −2.79(−2.9-−2.67) | 83.81(64.71-106.08) | −24.36 | −2.73(−2.81-−2.66) |
| Mozambique | 2.63(1.92-3.41) | 19.99 | −1.24(−1.4-−1.07) | 126(94.61-163.24) | 62.25 | −0.83(−0.94-−0.73) |
| Myanmar | 2.38(1.84-3.1) | −35.09 | −3.41(−3.65-−3.17) | 103.49(78.01-134.17) | −37.41 | −3.19(−3.4-−2.99) |
| Namibia | 2.19(1.6-2.85) | −29.34 | −3.6(−3.82-−3.38) | 110.84(81.97-145.28) | −3.04 | −2.62(−2.76-−2.47) |
| Nauru | 10.62(7.23-14.03) | −16.84 | −0.9(−1.45-−0.34) | 427.17(277.06-577.42) | −14.14 | −0.75(−1.35-−0.16) |
| Nepal | 13.36(8.76-20.49) | 11.96 | −2.12(−2.35-−1.89) | 358.56(240.44-506.56) | −8.1 | −2.56(−2.8-−2.31) |
| Netherlands | 0.8(0.65-0.97) | 48.22 | −0.92(−1.72-−0.12) | 13.02(11.14-15.19) | 6.14 | −1.87(−2.5-−1.23) |
| New Zealand | 1.92(1.65-2.2) | −1.47 | −2.77(−3.03-−2.51) | 48.57(42.45-55.08) | −23.36 | −3.04(−3.23-−2.84) |
| Nicaragua | 0.63(0.5-0.76) | −29.95 | −4.4(−4.75-−4.04) | 60.36(42.3-85.2) | 13.1 | −2.32(−2.44-−2.2) |
| Niger | 3.92(2.62-6.48) | 16.28 | −2.64(−2.79-−2.49) | 146.18(102.84-217.06) | 33.3 | −2.34(−2.51-−2.18) |
| Nigeria | 2.04(1.48-3.09) | −38.36 | −4.39(−4.6-−4.18) | 96.26(70.9-133.38) | 6.67 | −3.14(−3.33-−2.94) |
| Niue | 4.81(2.92-6.67) | −54.63 | −2.43(−2.51-−2.35) | 185.01(115.59-266.52) | −53.78 | −2.24(−2.34-−2.14) |
| North Macedonia | 1.26(0.98-1.59) | −51.86 | −4.95(−5.29-−4.62) | 33.79(26.4-42.45) | −57.62 | −5.27(−5.63-−4.91) |
| Northern Mariana Islands | 2.84(2.31-3.48) | −4.54 | −1.2(−1.39-−1) | 124.28(101.65-155.65) | −25.15 | −0.93(−1.08-−0.78) |
| Norway | 0.75(0.64-0.84) | −40.16 | −3.96(−4.49-−3.43) | 12.03(10.81-13.34) | −48.24 | −4.29(−4.78-−3.8) |
| Oman | 0.6(0.49-0.72) | −38.45 | −3.85(−4.05-−3.66) | 12.93(10.81-15.25) | −37.44 | −4.18(−4.29-−4.08) |
| Pakistan | 16.9(12.76-21.44) | 39.07 | −1.59(−1.88-−1.29) | 542.04(407.1-691.78) | 57.39 | −1.46(−1.75-−1.16) |
| Palau | 3.58(2.77-4.45) | −7.48 | −1.86(−2.01-−1.72) | 139.99(106.73-174.74) | −16.02 | −1.63(−1.78-−1.48) |
| Palestine | 0.74(0.58-0.89) | −20.31 | −3.94(−4.17-−3.7) | 41.6(31.01-54.86) | 47.75 | −2.53(−2.62-−2.44) |
| Panama | 0.62(0.44-0.85) | −38.95 | −4.88(−5.08-−4.68) | 62.3(44.4-85.1) | −8.19 | −2.72(−2.86-−2.59) |
| Papua New Guinea | 18.22(10.36-30.5) | 111.39 | −0.51(−0.64-−0.38) | 689(415.05-1081.71) | 114.37 | −0.42(−0.57-−0.28) |
| Paraguay | 0.87(0.61-1.13) | 22.7 | −1.91(−2.04-−1.79) | 68.16(49.69-90.54) | 45.41 | −1.04(−1.11-−0.96) |
| Peru | 0.66(0.45-0.91) | −20.62 | −4(−4.18-−3.82) | 56.28(39.39-78.3) | 8.88 | −1.97(−2.11-−1.82) |
| Philippines | 2.33(1.82-2.79) | 195.08 | 1.91(1.21-2.62) | 126.91(102.28-149.99) | 178.65 | 2.42(1.70-3.14) |
| Poland | 1.4(1.17-1.64) | −73.67 | −6.66(−7.16-−6.16) | 34.45(29.2-40.12) | −79.98 | −7.23(−7.8-−6.66) |
| Portugal | 1.14(0.96-1.35) | −30.45 | −3.94(−4.15-−3.74) | 23.69(20.36-27.7) | −55.05 | −4.84(−5.11-−4.57) |
| Puerto Rico | 0.46(0.34-0.6) | −33 | −3.9(−4.29-−3.51) | 14.22(10.72-18.59) | −43.55 | −3.58(−3.98-−3.18) |
| Qatar | 0.83(0.64-1.05) | 42.88 | −4.64(−4.86-−4.42) | 18.11(14.08-23.34) | 42.73 | −5.24(−5.43-−5.06) |
| Republic of Korea | 0.38(0.31-0.46) | 0.6 | −3.71(−4.08-−3.33) | 8.2(6.99-9.5) | −36.05 | −4.31(−4.57-−4.04) |
| Republic of Moldova | 1.4(1.13-1.75) | −75.15 | −6.08(−6.63-−5.52) | 52.25(42.36-64.76) | −78.21 | −6.35(−6.86-−5.84) |
| Romania | 1.18(0.94-1.47) | −73.28 | −6.38(−6.72-−6.03) | 33.36(26.61-40.98) | −80.46 | −6.94(−7.35-−6.53) |
| Russian Federation | 1.24(1.05-1.44) | −72 | −6.26(−6.76-−5.75) | 40.82(34.78-47.18) | −74.83 | −6.6(−7.11-−6.09) |
| Rwanda | 2.28(1.5-3.12) | −47.53 | −4.77(−5.11-−4.43) | 114.22(81.87-151.34) | −23.38 | −3.85(−4.14-−3.56) |
| Saint Kitts and Nevis | 1.01(0.7-1.34) | −52.54 | −4.48(−4.75-−4.21) | 33.4(21.03-46.14) | −56.32 | −4.84(−5.16-−4.52) |
| Saint Lucia | 1.74(1.37-2.2) | −24.65 | −3.96(−4.29-−3.62) | 100.5(77.94-128.89) | −17.35 | −2.67(−2.93-−2.42) |
| Saint Vincent and the Grenadines | 1.74(1.4-2.14) | −24.51 | −2.73(−2.92-−2.53) | 105.3(82.13-131.71) | −25.48 | −2.05(−2.19-−1.9) |
| Samoa | 7.63(5.43-10.32) | 0.06 | −1.48(−1.59-−1.37) | 300.75(208.11-412.42) | −0.31 | −1.16(−1.23-−1.09) |
| San Marino | 2.19(1.48-3.1) | 67.5 | −0.86(−1.09-−0.63) | 38.68(26.03-55.95) | 39.37 | −0.89(−1.12-−0.65) |
| Sao Tome and Principe | 4.49(2.72-6.34) | −9.61 | −2.07(−2.29-−1.84) | 153.04(108.38-204.68) | 8.13 | −1.84(−2.07-−1.62) |
| Saudi Arabia | 0.91(0.64-1.19) | −8.37 | −3.9(−4.2-−3.61) | 29.83(21.26-39.19) | −11.45 | −4(−4.3-−3.7) |
| Senegal | 2.27(1.64-3) | −8.65 | −2.66(−2.9-−2.42) | 100.43(73.92-131.01) | 15.13 | −1.92(−2.14-−1.7) |
| Serbia | 0.94(0.75-1.17) | −43.84 | −4.07(−4.41-−3.73) | 20.22(16.41-24.63) | −52.34 | −4.43(−4.78-−4.08) |
| Seychelles | 0.84(0.71-0.99) | −68.64 | −5.23(−5.93-−4.52) | 50.79(39.92-65.18) | −52.2 | −3.74(−4.32-−3.15) |
| Sierra Leone | 2.86(1.88-3.95) | −10.68 | −2.26(−2.4-−2.12) | 121.25(86.9-161.15) | 22.34 | −1.69(−1.82-−1.55) |
| Singapore | 0.34(0.28-0.42) | −54.58 | −6.51(−6.85-−6.17) | 10.1(8.46-12.16) | −62.86 | −6.75(−7.13-−6.37) |
| Slovakia | 1.06(0.83-1.34) | −26.8 | −2.44(−2.66-−2.22) | 29.4(23.25-36.36) | −31.86 | −2.65(−2.88-−2.43) |
| Slovenia | 2.08(1.54-2.82) | −1.77 | −2.49(−2.63-−2.35) | 37.59(28.76-49.44) | −31.77 | −3.38(−3.49-−3.28) |
| Solomon Islands | 21.09(10.94-31.16) | 55.5 | −1.1(−1.22-−0.98) | 809.52(404.45-1227.93) | 55.99 | −1.08(−1.2-−0.96) |
| Somalia | 4.95(2.79-8.83) | 72.85 | −1.56(−1.62-−1.51) | 201.86(129.94-315.23) | 103.25 | −1.33(−1.39-−1.27) |
| South Africa | 1.41(1.27-1.54) | −28.61 | −3.25(−3.82-−2.68) | 95.15(73.84-123.7) | −13.73 | −2.5(−2.9-−2.1) |
| South Sudan | 2.24(1.3-3.53) | −17.27 | −2.02(−2.22-−1.81) | 118.34(79.99-167.84) | 6.84 | −1.38(−1.56-−1.2) |
| Spain | 2.1(1.7-2.54) | −7.39 | −3.17(−3.29-−3.05) | 35.4(29.88-41.57) | −38.52 | −4.12(−4.27-−3.97) |
| Sri Lanka | 0.83(0.58-1.11) | −47.35 | −4.46(−4.62-−4.3) | 26.12(19.18-34.3) | −56.07 | −4.7(−4.92-−4.48) |
| Sudan | 2.7(1.76-4.2) | −37.45 | −3.25(−3.33-−3.18) | 108.54(74.99-156.32) | −34.82 | −3.28(−3.35-−3.21) |
| Suriname | 1.03(0.81-1.26) | −10.43 | −2.95(−3.19-−2.71) | 78.51(60.65-99.39) | 2.51 | −1.84(−2.01-−1.66) |
| Sweden | 0.75(0.59-0.91) | −36.94 | −2.9(−3.06-−2.73) | 12.02(10.23-14.03) | −47.08 | −3.33(−3.51-−3.14) |
| Switzerland | 0.66(0.53-0.79) | −55.98 | −5.29(−5.6-−4.99) | 11.32(9.59-13.11) | −60.42 | −5.14(−5.44-−4.84) |
| Syrian Arab Republic | 1.66(1.17-2.25) | −74.43 | −8.28(−9.33-−7.22) | 78.17(59.56-100.32) | −73.67 | −7.36(−8.32-−6.38) |
| Taiwan  (Province of China) | 0.63(0.46-0.82) | −60.8 | −7.77(−8.48-−7.06) | 19.97(15.44-25.33) | −58.23 | −6.39(−7.02-−5.76) |
| Tajikistan | 3.56(2.87-4.43) | −23.19 | −3.5(−3.83-−3.17) | 157.11(128.42-193.99) | −13.55 | −3.58(−3.84-−3.33) |
| Thailand | 0.5(0.37-0.66) | −75.99 | −9.55(−10.48-−8.61) | 38.86(28.81-52.06) | −66.16 | −6.51(−7.27-−5.74) |
| Timor-Leste | 3.07(2.05-4.38) | 30.8 | −1.76(−1.96-−1.56) | 133.79(87.53-185.56) | 23.54 | −1.57(−1.78-−1.35) |
| Togo | 2.64(1.85-3.73) | 13.25 | −2.55(−2.72-−2.38) | 109.55(81.36-144.9) | 37 | −1.87(−2.02-−1.72) |
| Tokelau | 6.51(4.66-8.84) | −55.84 | −2.82(−2.84-−2.79) | 242.3(168.59-334.88) | −55.1 | −2.69(−2.75-−2.62) |
| Tonga | 2.97(2.31-3.83) | −20.62 | −1.88(−1.97-−1.78) | 141.98(111.36-178.91) | −20.01 | −1.43(−1.55-−1.31) |
| Trinidad and Tobago | 0.89(0.63-1.23) | −48.2 | −4.66(−5.11-−4.21) | 73.93(53.96-98.35) | −35.97 | −2.79(−3.1-−2.48) |
| Tunisia | 1.06(0.7-1.48) | −13.27 | −3.01(−3.14-−2.87) | 28.33(19.63-39.06) | −40.58 | −3.61(−3.75-−3.48) |
| Turkey | 0.59(0.46-0.73) | −22.26 | −3.87(−4.31-−3.44) | 15.2(12.23-18.7) | −35.83 | −4.25(−4.66-−3.83) |
| Turkmenistan | 2.35(1.75-3.11) | −25.75 | −4.06(−4.54-−3.58) | 129.67(100.94-164.66) | −19.18 | −3.32(−3.74-−2.9) |
| Tuvalu | 10.69(7.23-15.32) | −33.41 | −2.34(−2.44-−2.24) | 393.67(266.02-573.41) | −37.17 | −2.36(−2.46-−2.26) |
| Uganda | 1.98(1.49-2.54) | 14.05 | −2.7(−2.92-−2.48) | 116.84(86.24-159.67) | 84.17 | −1.61(−1.81-−1.41) |
| Ukraine | 1.51(1.19-1.91) | −36.8 | −2.57(−3.25-−1.88) | 54.5(43.13-68.12) | −34.7 | −2.26(−2.94-−1.58) |
| United Arab Emirates | 3.45(2.08-5.28) | 219.33 | −3.6(−3.9-−3.29) | 108.7(71.93-156.08) | 254.82 | −3.09(−3.22-−2.97) |
| United Kingdom | 0.93(0.83-1.01) | −52.7 | −4.45(−4.69-−4.21) | 17.6(16.33-18.96) | −60.66 | −4.78(−5.05-−4.51) |
| United Republic of Tanzania | 1.91(1.44-2.41) | 6.47 | −2.39(−2.48-−2.3) | 107.78(80.39-143.54) | 48.52 | −1.45(−1.52-−1.38) |
| United States of America | 1(0.89-1.09) | −21.93 | −3.17(−3.53-−2.81) | 23.81(21.5-26.47) | −29.75 | −3.29(−3.71-−2.87) |
| United States Virgin Islands | 0.79(0.62-0.99) | −22.03 | −2.64(−2.82-−2.46) | 27.62(21.46-35.31) | −41.11 | −3.08(−3.31-−2.85) |
| Uruguay | 1.23(1.01-1.44) | −14.41 | −2.17(−2.36-−1.97) | 29.77(25.14-34.62) | −29.31 | −2.31(−2.55-−2.07) |
| Uzbekistan | 5.79(4.54-7.15) | 26.89 | −2.15(−2.65-−1.65) | 240.27(192.86-297.3) | 16.98 | −2.62(−3.11-−2.14) |
| Vanuatu | 15.96(10.09-23.97) | 98.69 | −0.92(−1.02-−0.81) | 627.15(410.43-941.07) | 98.78 | −0.71(−0.84-−0.58) |
| Venezuela  (Bolivarian Republic of) | 0.61(0.44-0.82) | −25.48 | −4.63(−5.09-−4.17) | 20.78(15.22-28.14) | −41.25 | −4.88(−5.42-−4.34) |
| Viet Nam | 1.8(1.22-2.29) | −23.18 | −3.71(−3.92-−3.49) | 47.27(34.55-59.54) | −26.8 | −3.66(−3.91-−3.42) |
| Yemen | 4.04(2.67-6.09) | 20.57 | −2.57(−2.75-−2.38) | 148.06(101.86-212.73) | 20.65 | −2.4(−2.53-−2.27) |
| Zambia | 2.76(1.97-3.5) | 18.47 | −1.7(−1.85-−1.55) | 126.26(97.39-162.71) | 42.33 | −1.58(−1.69-−1.48) |
| Zimbabwe | 5.71(3.79-7.76) | 62.25 | 0.89(0.47-1.32) | 261.38(187.9-340.64) | 60.18 | 1.00(0.61-1.40) |

RHD: rheumatic heart disease; EAPC: estimated annual percentage change; ASR, age-standardized rate; CI, confidence interval; UI: uncertainty interval; DALYs, disability-adjusted life years.

**Supplementary table 4.** The number and age-standardized rate of death and DALYs due to RHD in attributable risk factors globally, in both sexes, in 1990 and 2019, and percentage change in absolute number and the EAPCs from 1990 to 2019.

|  | **1990** | | **2019** | | **1990-2019** | |
| --- | --- | --- | --- | --- | --- | --- |
| **Characteristics** | Number  ×10^3^ (95% UI) | ASR/100,000  (95% UI) | Number  ×10^3^ (95% UI) | ASR/100,000  (95% UI) | Percentage (%) | EAPC  (95%CI) |
| **Death related risks** |  |  |  |  |  |  |
| Environmental/occupational risk | 9.39(5.10-16.10) | 0.23(0.12-0.41) | 7.69(4.09-13.50) | 0.10(0.05-0.17) | −18.11 | −3.03(−3.14-−2.92) |
| Metabolic risk | 84.03(55.99-127.73) | 2.12(1.40-3.27) | 76.41(50.97-114.75) | 0.95(0.63-1.45) | −9.07 | −2.85(−2.89-−2.81) |
| Behavioral risk | 19.14(6.37-42.26) | 0.47(0.15-1.05) | 11.92(2.78-29.07) | 0.15(0.03-0.36) | −37.73 | −3.99(−4.06-−3.92) |
| **DALYs related risks** |  |  |  |  |  |  |
| Environmental/occupational risk | 320.20(168.87-543.63) | 7.18(3.87-12.16) | 213.51(106.84-372.74) | 2.58(1.28-4.55) | −33.32 | −3.53(−3.66-−3.40) |
| Metabolic risk | 2677.38(1784.62-3903.96) | 61.19(41.08-89.77) | 2446.38(1672.9-3445.31) | 29.72(20.33-41.9) | −8.63 | −2.61(−2.66-−2.57) |
| Behavioral risk | 624.13(213.07-1323.87) | 14.24(4.81-30.28) | 390.75(98.06-930.51) | 4.71(1.18-11.19) | −37.39 | −3.83(−3.91-−3.76) |

RHD: rheumatic heart disease; EAPC: estimated annual percentage change; ASR, age-standardized rate; CI, confidence interval; UI: uncertainty interval.

**Supplementary table 5.** The number and age-standardized rate of death and DALYs due to RHD in attributable risk factors globally, in sexes, in 2019, and percentage change in number and the EAPCs from 1990 to 2019.

|  | **Male** | | | **Female** | | |
| --- | --- | --- | --- | --- | --- | --- |
| **Characteristics** | ASR/100,000  (95% UI) | Percentage change (%) | EAPC  (95%CI) | ASR per 100k  (95% UI) | Percentage change (%) | EAPC  (95%CI) |
| **Death related risks** |  |  |  |  |  |  |
| Environmental/occupational risk | 0.10(0.05-0.18) | −22.48 | −3.13(−3.26—3.00) | 0.09(0.04-0.16) | −13.39 | −2.93(−3.02-−2.83) |
| Metabolic risk | 0.88(0.58-1.35) | −4.52 | −2.59(−2.63-−2.54) | 1.01(0.65-1.55) | −12.18 | −3.04(−3.09-−2.98) |
| Behavioral risk | 0.16(0.04-0.38) | −29.89 | −3.51(−3.58-−3.43) | 0.13(0.02-0.35) | −44.51 | −4.50(−4.59-−4.41) |
| **DALYs related risks** |  |  |  |  |  |  |
| Environmental/occupational risk | 2.69(1.38-4.61) | −36.94 | −3.64(−3.80-−3.47) | 2.50(1.15-4.62) | −29.28 | −3.42(−3.53-−3.31) |
| Metabolic risk | 28.31(18.53-41.02) | −3.03 | −2.30(−2.35-−2.25) | 30.96(20.89-44.89) | −12.94 | −2.87(−2.95-−2.79) |
| Behavioral risk | 5.16(1.43-11.59) | −29.18 | −3.31(−3.38-−3.24) | 4.29(0.83-11.15) | −44.69 | −4.39(−4.50-−4.28) |

RHD: rheumatic heart disease; EAPC: estimated annual percentage change; ASR, age-standardized rate; CI, confidence interval; UI: uncertainty interval; DALYs, disability-adjusted life years.

**Supplementary table 6**. The EAPCs of death and DALYs due to RHD in attributable risk factors in SDI quintiles from 1990 to 2019.

|  | **Low SDI** | **Low-middle SDI** | **Middle SDI** | **High-middle SDI** | **High SDI** |
| --- | --- | --- | --- | --- | --- |
| **Characteristics** | EAPC (95%CI) | EAPC (95%CI) | EAPC (95%CI) | EAPC (95%CI) | EAPC (95%CI) |
| **Death related risks** |  |  |  |  |  |
| Environmental/occupational risk | −1.75(−1.94-−1.56) | −2.68(−2.84-−2.52) | −4.25(−4.36-−4.14) | −4.54(−4.73-−4.35) | −3.69(−3.78-−3.61) |
| Metabolic risk | −1.66(−1.77-−1.55) | −2.17(−2.27-−2.07) | −3.52(−3.58-−3.45) | −4.6(−4.77-−4.43) | −3.96(−4.24-−3.67) |
| Behavioral risk | −1.61(−1.69-−1.53) | −2.91(−2.95-−2.87) | −5.06(−5.18-−4.95) | −5.28(−5.40-−5.16) | −3.79(−4.04-−3.55) |
| **DALYs related risks** |  |  |  |  |  |
| Environmental/occupational risk | −2.33(−2.55-−2.11) | −3.29(−3.47-−3.11) | −5.01(−5.12-−4.9) | −5.35(−5.56-−5.15) | −4.65(−4.77-−4.54) |
| Metabolic risk | −1.55(−1.64-−1.46) | −2.11(−2.21—2.00) | −3.34(−3.40-−3.29) | −4.64(−4.81-−4.47) | −4.34(−4.70-−3.97) |
| Behavioral risk | −1.55(−1.64-−1.46) | −2.76(−2.81-−2.72) | −5.03(−5.14-−4.93) | −5.09(−5.21-−4.97) | −4.15(−4.46-−3.85) |

RHD: rheumatic heart disease; EAPC: estimated annual percentage change; ASR, age-standardized rate; CI, confidence interval; UI: uncertainty interval.
